# Paratope mapping of tilvestamab, an anti-AXL function-blocking antibody, using high-throughput bacterial expression of secreted scFv-osmY fusion proteins

**DOI:** 10.1042/BSR20253747

**Published:** 2025-10-28

**Authors:** Eleni Christakou, Petri Kursula, David Micklem

**Affiliations:** 1 Department of Biomedicine, University of Bergen, Bergen, 5009, Norway; 2 BerGenBio ASA, Bergen, 5009, Norway; 3 Faculty of Biochemistry and Molecular Medicine & Biocenter Oulu, University of Oulu, Oulu, 90014, Finland; 4 LINXS Institute of Advanced Neutron and X-Ray Science, Lund, 22484, Sweden

**Keywords:** antibodies, protein binding, protein conformation, receptor tyrosine kinases, recombinant proteins

## Abstract

Targeting AXL receptor kinase with a highly selective antibody presents a promising approach for inhibiting AXL and potentially improving cancer treatment. An essential step in antibody optimisation is the mapping of paratope residues to epitope residues. In the present study, we identify the residues of tilvestamab, a function-blocking anti-AXL monoclonal antibody, that are essential for its binding to the extracellular domain of AXL. A single-chain variable fragment (scFv) fused to osmotically inducible protein Y (osmY) was designed to enable the secretion of soluble scFv-osmY mutants, which could be directly subjected to high-throughput biolayer interferometry screening for binding to the AXL Ig1 domain. Each complementarity-determining region residue of scFv was mutated to Ala, while additional mutations were made on the basis of predicted contribution to binding. We generated AlphaFold3 predictions for the scFv (tilvestamab)-AXL Ig1 complex to gain insights into the molecular interactions of the essential residues, as determined by the experimental data. Our study reveals that tilvestamab binds to the Ig1 domain of AXL, with twelve residues on scFv (tilvestamab) contributing most to binding, likely being situated at the binding interface. Glu2 near the N-terminus of AXL is essential for binding. The data give a structural view into the AXL-tilvestamab complex and allow for further optimisation of the binding interface.

## Introduction

The transmembrane protein AXL (from the Greek word anexelekto) belongs to the TAM (TYRO3, AXL and MERTK) family of receptor tyrosine kinases and regulates a wide range of physiological cellular functions essential for cell survival, proliferation and migration [1]. AXL is composed of an extracellular domain, a single transmembrane domain and a cytosolic tyrosine kinase domain. The N-terminal extracellular domain interacts with extracellular ligands to initiate signal transduction. This ectodomain consists of two immunoglobulin-like (Ig-like) domains and two fibronectin type III domains. A single transmembrane domain is followed by an intracellular domain harbouring tyrosine residues conserved among the TAM receptors, as well as the kinase domain. The Ig domains within the ectodomain of AXL interact with its primary ligand, growth arrest-specific protein 6 (GAS6), leading to receptor dimerisation, autophosphorylation of the tyrosine residues and activation of the intracellular signalling cascades [2].

AXL expression is up-regulated in numerous human malignancies, such as leukaemia, breast cancer, prostate cancer [3], kidney cancer [4], melanoma [5], glioblastoma [6] and non-small lung cancer [7], and it is associated with cancer progression, therapy resistance and poor prognosis [8–11]. Given its role in cancer development, AXL has emerged as a promising prognostic marker and therapeutic target. AXL-targeted therapies with small-molecule inhibitors, which target the kinase domain in either its active or inactive conformation, have shown promising results and have progressed into various phases of clinical investigation [12–14]. Antibody therapies present an alternative approach to small-molecule inhibitors with higher selectivity, thereby minimising off-target toxicity. High-affinity anti-AXL monoclonal antibodies (mAbs) exert tumour inhibition either as a single agent or in combination with known chemotherapeutic drugs [15].

Tilvestamab (BGB149) is a function-blocking, humanised and highly selective anti-AXL mAb developed by BerGenBio [16]. In the present study, we investigated the essential amino acids that are involved in the interaction of tilvestamab with its target antigen, AXL. The variable V_H_ and V_L_ domains of antibodies each contain three hypervariable loops, known as complementarity-determining regions (CDRs), which pack together at the surface of the antibody to form the paratope that binds to the antigen epitope. To map the paratope of tilvestamab, we generated a panel of bacterially expressed single-chain variable fragments (scFvs) carrying tilvestamab CDRs with various single point mutations and screened them for their ability to bind the Ig1 domain of human AXL. The set of point mutations included mutation of each CDR residue to alanine and additional mutations of key residues (as defined in [17]) to a wider set of substitutions. To enable rapid functional screening of the antibody fragments, the scFvs were fused to the secreted bacterial osmotically inducible protein Y (osmY), allowing testing of protein directly in the culture supernatant [18]. Finally, we demonstrate how the paratope, defined by the experimental data, aligns to the AlphaFold structural prediction. The updated AlphaFold3 exhibits higher accuracy in modelling antibody–antigen complexes compared with its predecessor, AlphaFold2 [19]. The 3D structural model predicted using AlphaFold3 reveals how the key amino acids identified in the screen interact at the molecular level, highlighting the specific binding interfaces that drive the formation of the antibody–antigen complex. Taken together, the results can assist the design of second-generation therapeutic antibodies with improved properties.

## Materials and methods

### Design of plasmid encoding scFv-osmY fusion protein

The sequence of tilvestamab was reconfigured into a shorter scFv antibody format, incorporating the variable domains of the heavy (V_H_) and the light (V_L_) chains, joined together by a short flexible peptide linker AKTTPPKLEEGEFSEARV (adapted from the vector pOPE101, Progen, Genbank accession Y14585). The scFv was connected by a short linker, RADAAPTVSAA, to the gene coding for the bacterial osmY. This was followed by a Myc-tag (GSEQKLISEEDL), facilitating detection of the fusion protein by the mouse mAb Myc1-9E10, and a His_6_-tag. The sequence was synthesised and cloned into the bacterial T7-driven expression vector pET-22b (+) by Genscript. All plasmid DNA sequencing was carried out at Eurofins Genomics.

All protein sequences and primers used are listed in the supplementary material. The scFv-osmY fusion protein expression system allows for high-throughput, low-effort expression of scFv secreted directly into the medium (not just the periplasmic space) [18].

### Mutagenesis

scFvs were diversified by mutagenesis as described in the Q5 Site-Directed Mutagenesis Kit (New England Biolabs). Mixtures of mutagenic primers were prepared at 1 µM for each primer. Plasmid template (pET-22b-osmY-scFv) was diluted to 5 ng/µl in water and then to 50 pg/µl in 2X Q5 Mastermix. Then, in a PCR plate, 4 µl of the master mix and 4 µl of the appropriate primer mix were distributed to each well. Reactions were run in the following thermal cycler programme: 98 °C for 30 s; 24 cycles (98 °C for 10 s; anneal for 10 s; 72 °C for 210 s); 4 °C hold. The annealing temperature was chosen according to the primer pair, using a gradient on the thermocycler when appropriate. Products were treated with the KLD (kinase, ligase, DpnI) mix to degrade the parental vector, add 5′ phosphates to the DNA products and ligate the linear DNA into circular plasmids. A master mix was made by adding 1/5 vol KLD enzyme mix to the 2 x KLD reaction buffer and distributed as 1 µl aliquots. 0.7 µl of each PCR product diluted 1+3 in water was added and mixed. After incubation for at least 5 min at room temperature, 5 µl of the KLD reaction was transformed into chemically competent T7 express *E. coli* cells via heat shock, plated on LB+carbenicillin plates and incubated at 37 °C overnight.

### Bacterial expression of tilvestamab scFv fragments

Autoinduction is a method to induce protein expression from T7-lacO-regulated promoters without requiring the addition of the expensive lactose analogue, isopropyl β-D-thiogalactopyranoside. It is based on the observation that *E. coli* will preferentially use glucose and glycerol as an energy source even in the presence of lactose and that these carbon sources will maintain lacO in an inactive form. Only when the glucose and glycerol have been used up (and when the culture is at a high density) will the lactose allow T7-mediated expression [20].

Autoinduction was accomplished by inoculating single colonies of a bacterial T7 expression strain carrying an scFv-osmY fusion protein construct (pET-22b-osmY-scFv or mutated variants) into 1 ml of autoinduction medium (LB broth supplemented with 0.5% glycerol, 0.05% glucose, 0.2% lactose, 50 µM Fe(III)(NO_3_)_3_, 20 µM CaCl_2_, 2 µM NiCl_2_, 2 µM CoCl_2_, 2 µM boric acid, 25 mM Na_2_HPO_4_, 25 mM KH_2_PO_4_, 25 mM (NH_4_)_2_SO_4_ and 100 µg/ml carbenicillin) and grown with vigorous shaking at 37 °C for at least 24 h. Bacteria were pelleted by centrifugation at top speed in a benchtop microcentrifuge. Pellets were retained for DNA preparation and sequencing, and the scFv-osmY fusion protein-containing supernatant was used directly in Western blotting and Octet binding experiments.

### Detection of scFv-osmY proteins by Western blotting

For each mutant, bacteria were pelleted, and 10–40 µl of bacterial supernatant was run on an SDS-PAGE MiniPROTEAN TGX stain-free 4–20% gel (BioRad) and blotted onto a polyvinylidene difluoride membrane (GE Healthcare Life Sciences). The membranes were blocked with 10 ml of 5% (w/v) skimmed milk in Tris-buffered saline/Tween-20 (TBS-T; 25 mM Tris-HCl, 1 mM NaCl, and 0.1% Tween-20) followed by overnight incubation at 4 °C in 5 ml of incubation buffer (TBS-T with 5% skimmed milk) containing biotinylated anti-Myc primary antibody (Millipore 16–170) in 1/1000 dilution. The membranes were then washed three times with PBS-T (0.01% Tween-20 in phosphate-buffered saline) and incubated with the secondary reagents HRP-Streptavidin (BD Pharmingen 51–75477E, further diluted 1/1000) and horseradish peroxidase (HRP)-StrepTactin (BioRad Cat#1610380, diluted 1/10,000) in 5 ml of incubation buffer for 20 min–4 h at room temperature. Finally, after three washes with PBS-T, protein bands were visualised with enhanced chemiluminescence (Pierce^TM^ ECL Substrate, 32106) and imaged using a ChemiDoc^TM^ XRS+ imager with Image Lab software (Biorad Hercules, CA).

### Biolayer interferometry screening of scFv-osmY supernatants

Mutated scFv-osmY were screened for binding to the Ig1-domain of human AXL by biolayer interferometry (BLI) on an Octet RED96 instrument (Pall ForteBio). Rat Axl-Fc was used as a control; see Supplementary Information for details on production of human and rat AXL-Fc fusions. Reagents and samples were dispensed into 96-well polypropylene, black flat-bottom microplates (Greiner). Dip and Read™ Ni-NTA coated biosensor tips (ForteBio) were used to capture the His-tagged scFv-osmY proteins directly from the undiluted bacterial supernatants. The method used consisted of the following steps: (1) equilibration of tips in kinetic buffer (0.02% Tween 20, 0.05% sodium azide, 0.1% BSA in PBS) to acquire baseline (120 s); (2) loading of scFv-osmY by transferring the biosensors into wells containing undiluted supernatant (600 s); (3) rinsing of tips (5 s) to remove non-specific proteins and baseline acquisition in kinetic buffer (120 s); (4) association of AXL Ig1 domain Fc (5.6 μg/ml in kinetic buffer); (5) dissociation by dipping the biosensors into wells containing kinetic buffer; (6) regeneration of biosensors by immersion in 10 mM glycine (pH 1.5); and neutralisation in kinetic buffer (3 * 5 s cycles); (7) recharging of biosensors with 10 mM NiCl_2_ (60 s).

**Figure 1:**
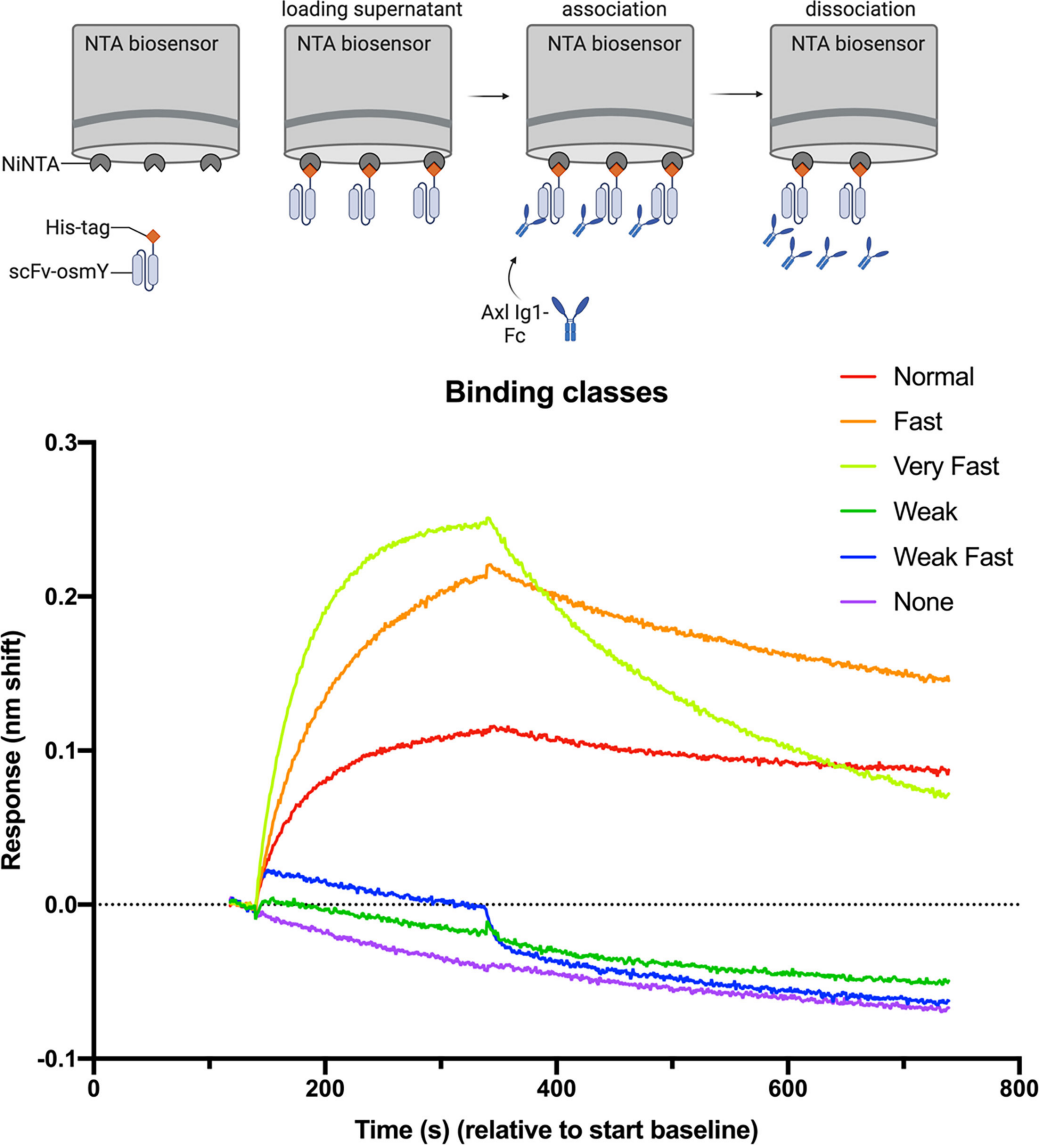
Schematic representation of the BLI strategy. Top: NTA biosensors were used to capture scFv-osmY mutants and test their binding to AXL Ig1-Fc. Bottom: the representative binding sensorgram shows different examples of binding curves from the BLI experiments and how the binding was classified as normal, fast, very fast, weak, weak fast and no binding.

Binding was analysed by isolating the association/dissociation phases and aligning them on the y-axis by subtraction of the baseline acquired in step 4. Individual BLI traces were scored by eye to determine whether the scFv-osmY being tested bound apparently normally to AXL-Ig1-Fc (normal), failed to bind at all (none), or showed an intermediate effect. ‘Fast’ and ‘Very Fast’ describe scFv-osmY strong initial binding during the association phase, followed by rapid or very rapid loss of signal (compared with the parental scFv-osmY). In addition, some scFv-osmY were scored as ‘Weak’ or ‘Weak Fast’; these showed detectable but weak initial binding followed by slow or rapid dissociation. Examples of binding curves are shown in Figure 1.

The strength of binding was not obviously related to the expression level of the scFv-osmY as determined by Western blotting. To visualise the binding information in a more condensed form, a ‘severity score’ was assigned to each binding conclusion to indicate how severely binding was affected. According to the scoring system, 0, 1, 2 or 3 points were assigned to normal, fast, very fast and weak binding, and the higher scores 4 and 5 indicated very weak or weak fast or no binding. Substitutions that were scored ‘normal’ or ‘fast’ were considered tolerated, while all other severities were considered not tolerated. The screen was performed ‘blind’, with binding assessed before sequencing revealed the identity of the mutation. 1–12 observations were made for each amino acid change, and the qualitative score was consistent across replicates.

### Protein structure prediction and interpretation

An AlphaFold3 model of the AXL-Ig1-scFv complex was generated at the AlphaFold3 server [19] (https://alphafoldserver.com/), using default parameters. Several independent runs through the course of the study gave essentially identical results. For comparison, an AlphaFold2 [21] model was made using the AlphaFold2 Colab [22] (https://colab.research.google.com/github/sokrypton/ColabFold/blob/main/AlphaFold2.ipynb). Default parameters were used, and the results were reproducible. Visualisation and analysis of the structures were carried out in PyMOL [23].

## Results and discussion

**Figure 2:**
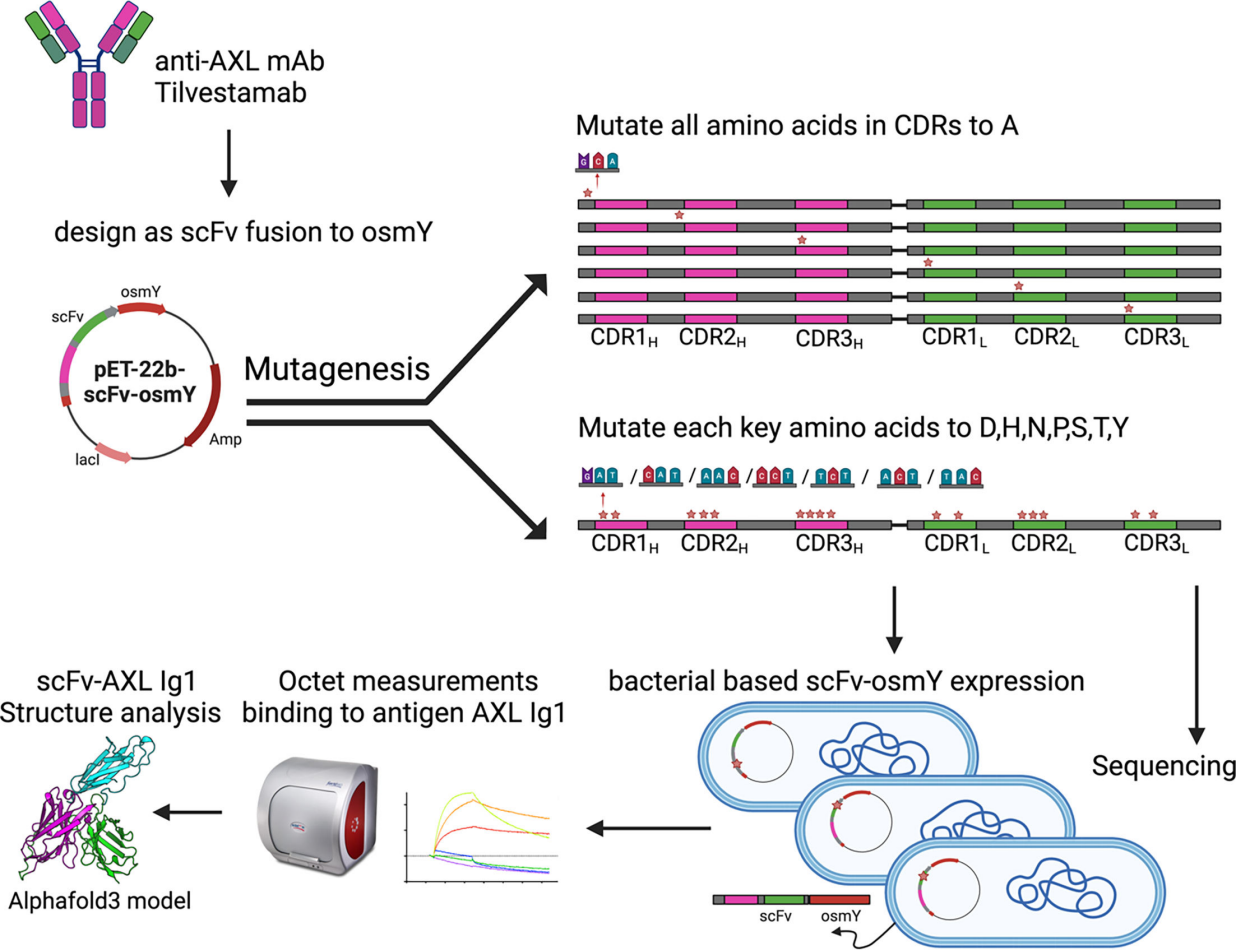
Overview of the experimental strategy for the identification of essential residues of tilvestamab for binding to AXL. Site-directed mutagenesis was used to alter each CDR residue to alanine, while additional mutations were also made on selected residues. BLI was used to test each mutant binding to the Ig1 domain of AXL. Finally, the experimental data were linked to AlphaFold3 prediction of the complex scFv-Ig1 AXL.

The identification of essential residues of tilvestamab for binding to the AXL Ig1 domain involved the following steps (illustrated in Figure 2). First, a scFv-osmY antibody-like protein was designed, carrying the variable regions of tilvestamab. Next, all residues within the CDR loops were mutated to alanine, while additional mutations were made on selected residues guided by the work of Robin et al., predicting certain residues to contribute most to the binding energy [17]. Mutants were sequenced and tested individually for binding to AXL Ig1-Fc protein by BLI. After experimentally scoring the mutations, an AlphaFold3 model of the complex was generated for structural insights.

### Tilvestamab expressed as a single-chain antibody fragment fused to osmY binds the AXL Ig1 domain

**Figure 3:**
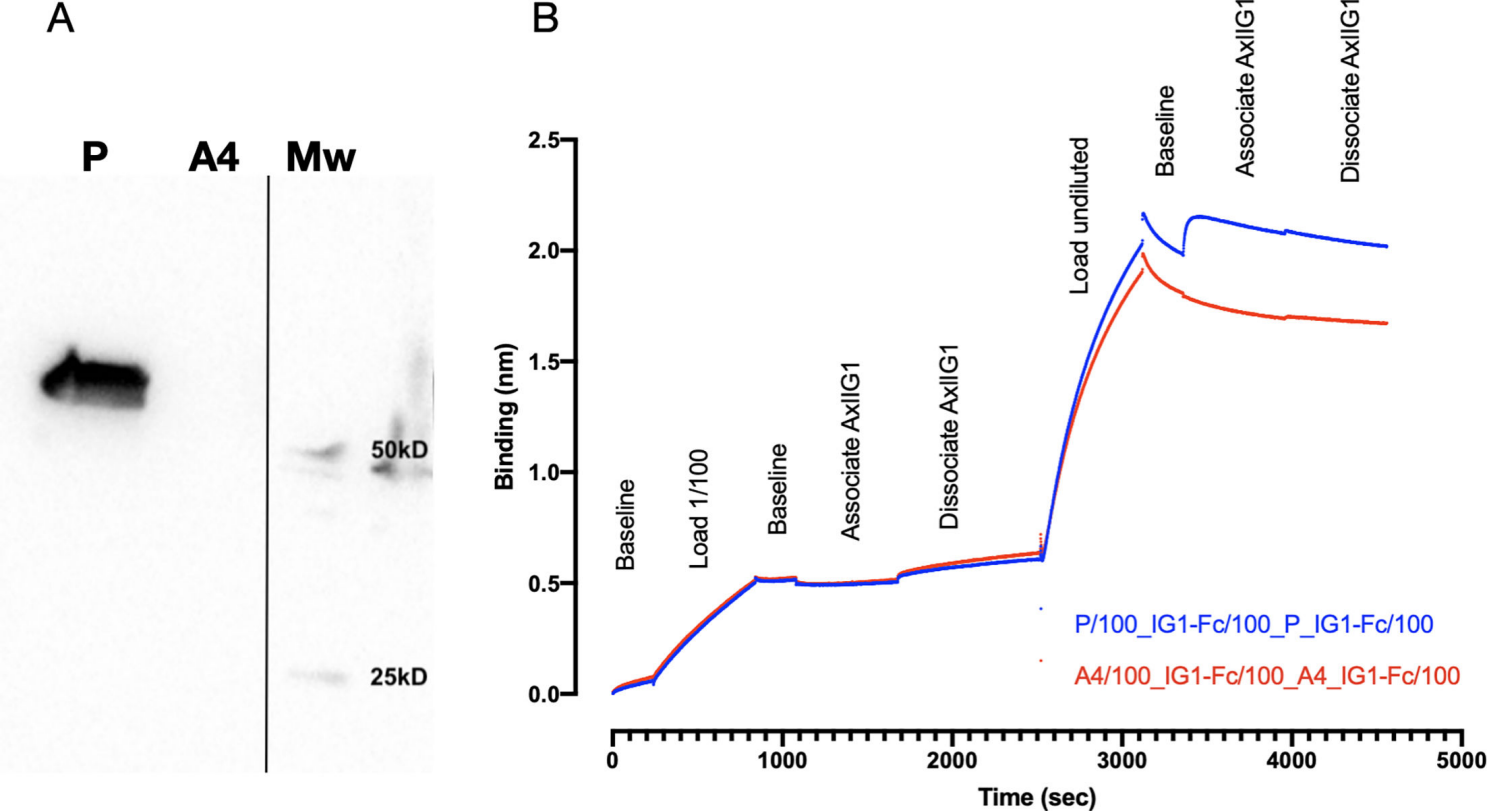
Parental scFv-osmY expressed in bacteria binds to AXL Ig1 domain. (**A**) Western blot with anti-Myc antibody detects a strong band in supernatant from bacterial cells expressing Parental scFv-osmY (**P**) but not from control supernatant from cells expressing a frame-shifted mutation (**A4**). Mw: Molecular weight markers. (**B**) Biolayer interferometry analysis on an Octet RED96 demonstrates binding of P (but not A4) from undiluted culture supernatant to human AXL IG1-domain-Fc fusion protein (‘Associate AXLIG1’). Supernatant used at 1/100 (first 2500 s) did not give a detectable response.

The CDRs of tilvestamab were reconfigured as a scFv fused to the bacterial protein osmY carrying Myc and His6 tags for high-throughput production of antibodies in *E. coli* (parental plasmid map, Supplementary Figure S1). The protein produced from this vector is referred to as ‘parental’. Following expression in a T7-expressing strain of *E. coli,* the approximately 52 kDa protein could be readily detected in the culture supernatant by Western blotting with an anti-Myc antibody (Figure 3A).

The His6-tag on the scFv-osmY fusion proteins allows them to associate with NiNTA-coated tips for BLI experiments (Figure 3B). Tips loaded with undiluted culture medium from cells expressing the parental tilvestamab-like scFv-osmY bound strongly to the AXL Ig1 domain fused to human Fc (blue, *t*=3300 s onwards, Figure 3B). Control supernatant (frameshift mutated scFv-osmY) demonstrated loading of unspecific proteins onto the tips (*t*=2500–3100 s), but no association with AXL Ig1 Fc (red, *t*=3300 s onwards). No specific interaction could be seen when culture supernatant was used diluted 1/100 (*t*=0–2500 s). As expected, parental scFv was unable to bind rat Axl Fc (Supplementary Figure S2). Thus, the parental scFv-osmY fusion protein based on the antibody recognition sites of tilvestamab retained the ability to specifically bind the human AXL Ig1 domain.

### Design of a panel of mutated scFv-osmY variants

**Figure 4:**
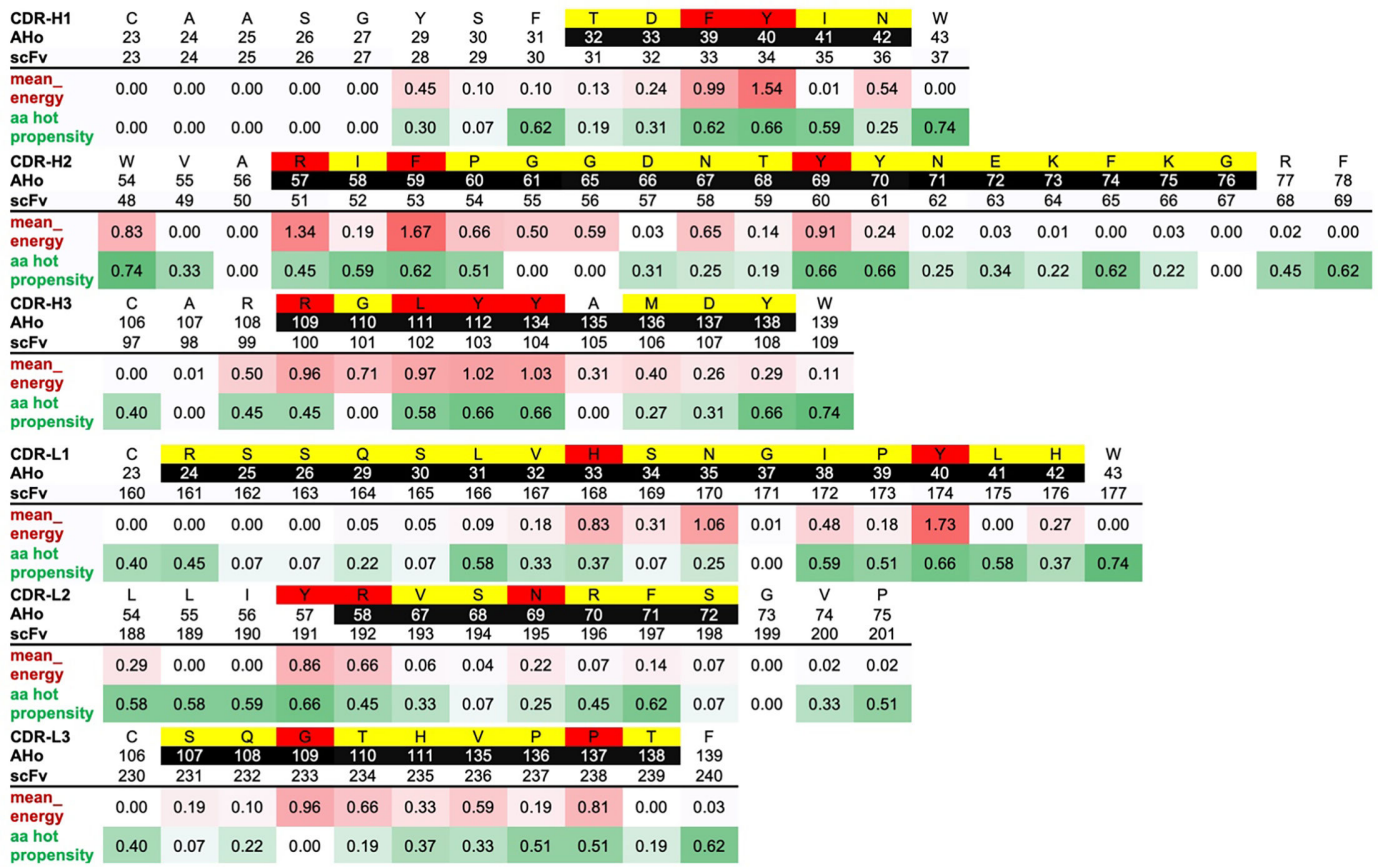
Mutations of heavy chain CDRs (CDR-H) and light chain CDRs (CDR-L) from tilvestamab. Tilvestamab seq: The sequence of each CDR and surrounding framework-region sequence. AHo (Honneger’s numbering scheme): the numbering of the amino acid according to the numbering scheme of Honegger and Plückthun [24]. CDR residues as defined by Kabat are shown as white text on black shading. scFv: numbering of the amino acid within the scFv-osmY fusion protein. mean_energy: the average binding free energy contributed by each site [17]. aa hot propensity: a measure of how often an amino acid makes an energetically favourable bond when it is in a hot-spot location in the sequence adapted from Robin et al. [17]. Amino acids targeted for mutation to alanine are shaded yellow. Amino acids targeted for mutation to ADHNPSTY are shaded red.

Robin et al. computationally identified the residues within antibody CDRs that are most likely to contribute energetically to antigen binding [17]. Using the worksheet that they published, we identified residues within the CDRs that are likely to contribute to binding (Figure 4). These residues were mutated using degenerate oligonucleotides with the appropriate codon replaced by NMT (AGCorT, AorC, T), which encodes an amino acid from the set alanine (A), aspartate (D), histidine (H), arginine (N), proline (P), serine (S), threonine (T) and tyrosine (Y), representing a broad subset of amino acid classes including charged (DH), polar (DHNSTY), hydrophobic (AHTY), hydrophilic (DNPS), aromatic (Y), tiny (SA) and small (ADNSP). In addition, a small number of mutations to leucine were generated. All other residues in the CDRs were individually mutated to alanine. The set of primers used for site-directed mutagenesis is listed in Supplementary Table1 and Supplementary Table 2.

It should be noted that the mutagenesis was strictly restricted to amino acids previously implicated in antigen binding (in and near the CDRs) [17] and is thus not expected to affect overall folding of the core Ig domain. All severity scores except ‘very weak’ and ‘no binding’ required observation of a binding signal, implying the presence of at least some folded antibody. However, we cannot distinguish for certain for each substitution whether a substitution that is not tolerated is due to loss/gain of a specific interaction with the antigen or loss of integrity of the scFv. We considered this approach, however, to be suitable for identifying binding interfaces in a protein–protein complex, without the need for scFv purification, and not aiming for atomic resolution of the interactions.

### Scoring scFv-osmY variants

**Figure 5:**
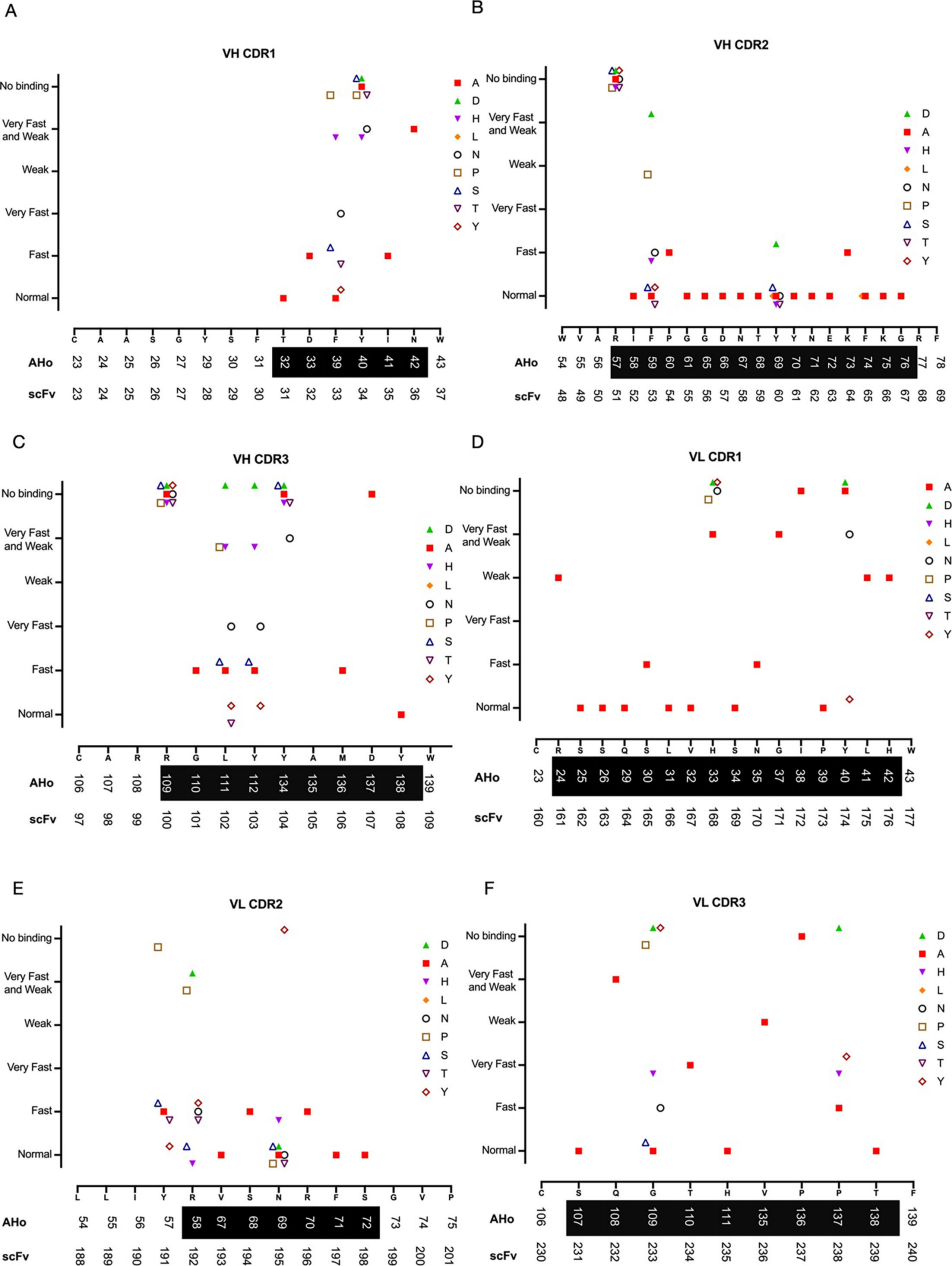
Effect of mutations binding to AXL Ig1-Fc for all six CDR loops of tilvestamab scFv. (**A**) heavy chain CDR1, (**B**) heavy chain CDR2, (**C**) heavy chain CDR3, (**D**) light chain CDR1, (**E**) light chain CDR2 and (**F**) light chain CDR3. The results are plotted with severity score on the y-axis vs. the original sequence on the x-axis. AHo numbering, the location of each CDR and the numbering of the amino acid within the scFv are also indicated. AHo: the numbering of the amino acid according to the numbering scheme of Honegger and Plückthun [24]. CDR residues as defined by Kabat are shown as white text on black shading. scFv: numbering of the amino acid within the scFv-osmY fusion protein.

scFv-osmY variants were expressed in *E. coli* and analysed using BLI. Each scFv-osmY variant protein was first loaded onto the NiNTA sensor tips, and the association and dissociation of human AXL Ig1-Fc protein to the immobilised scFv-osmY was then followed, leading to assignment of a severity score for each mutation. The BLI experiments represent a high-throughput screen of mutations, performed on bacterial culture supernatants, with the only purification occurring through the selective binding of the His_6_-tagged scFvs to the NiNTA Octet probes. As such, the concentration of scFv was unknown, making K_on_ and K_d_ impossible to assess. For this reason, the effects of the mutations were assessed only qualitatively, based on the dissociation phase, as K_off_ is independent of the concentration of the antigen–scFv complex. In Figure 5A–F, the results for each scFv-osmY variant are plotted. Overall, all six CDR loops of the tilvestamab scFv were important for the binding interaction, while for both the V_H_ and V_L_ chains, CDR2 appeared to tolerate the most mutations.

The results shown in Figure 5A suggest that the Tyr at AHo 40 (scFv Y34) and the Asn at AHo 42 (N36) are essential for binding. AHo 39 (scFv F33) tolerates the conservative substitution to Y and the small amino acids A, S and T but not P, H or N. T31, D32 and I35 all tolerate substitution to Ala, suggesting that they do not contribute greatly to binding energy.

The results shown in Figure 5B suggest that the Arg at AHo 57 (scFv R51) is essential for binding. Substitution to Ala has only a minor effect for all other amino acids in CDR2. AHo 59 (F53) tolerates most tested substitutions except for Pro and the negatively charged Asp.

The results shown in Figure 5C suggest that several amino acids in the V_H_-chain CDR3 are important for binding: the Arg at AHo 109 (scFv R100), the Tyr at AHo 134 (scFv Y104) and the Asp at AHo 137 (scFv D107) do not tolerate substitution to any of the tested amino acids. Substitution to Ala has only a minor effect for AHo 110 (scFv G101), AHo 136 (scFv M106) and AHo 138 (scFv Y108). AHo 111 (scFv L102) and AHo 112 (scFv Y103) tolerate only some substitutions. In both cases, substitutions to Ala, Ser or Tyr are tolerated, while substitution to Arg, His or Asp causes progressively worse binding. scFv L102 was also tested for substitution to Thr (tolerated) and Pro (not tolerated).

In the V_L_ chain CDR1 (Figure 5D), AHo residues 24, 33, 37, 38, 40, 41 and 42 (scFv R161, H168, G171, I172, Y174, L175 and H176) are all essential for strong binding and do not tolerate substitution to Ala or any other amino acid tested.

In the V_L_ chain CDR2 (Figure 5E), all CDR residues tolerate substitution to Ala (not tested for AHo 58, scFv R192). Most other substitutions are well tolerated apart from the AHo 57 (scFv Y191) mutation to Pro, the AHo 58 (scFv R192) mutation to Pro or Asp and the AHo 69 (scFv N195) mutation to Tyr.

Finally, in the V_L_ chain CDR3 (Figure 5F), four CDR residues do not tolerate substitution to Ala (AHo 108, 110, 135, 136; scFv Q232, T234, V236 and P237). In addition, AHo 109 and AHo 137 (G233 and P137) do not tolerate substitution to Asp, Tyr, His or Pro (G233 only), while tolerating Ala.

### Structural interpretation of the experimental data based on AlphaFold3 predictions

**Figure 6:**
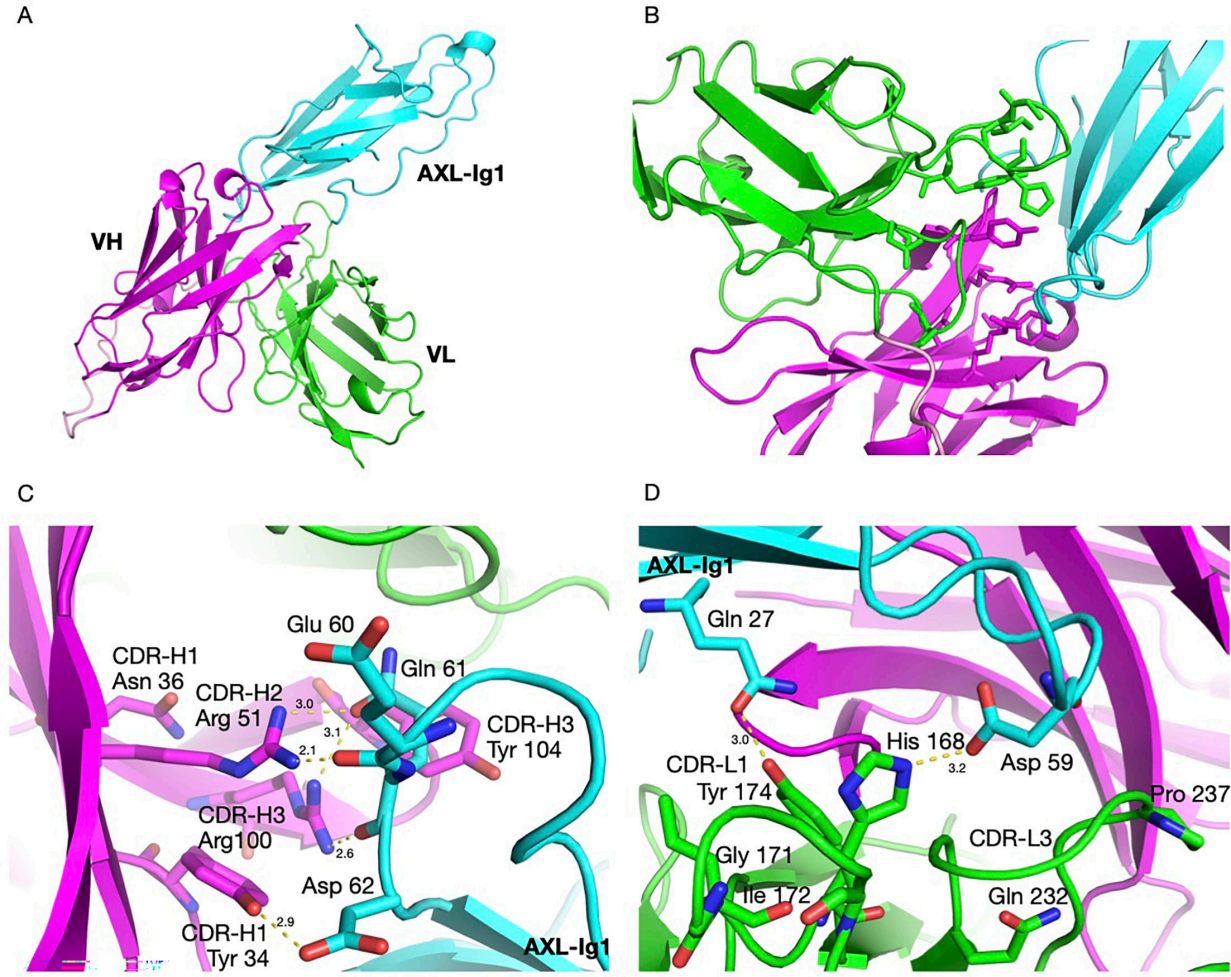
AlphaFold3 structure prediction and analysis. (**A**) Overall view of AXL Ig1-scFv complex. AXL Ig1 domain is indicated in cyan. The V_H_ and V_L_ of tilvestamab are coloured magenta and green, respectively (**B**) Tilvestamab paratope with the side chains of the 12 key amino acids as identified by the alanine-scanning experimental data. (**C,D**) Key amino acids interactions between AXL Ig1 and V_H_ domain (**C**) and V_L_ domain (**D**). Oxygen and nitrogen atoms of the side chains are coloured red and blue, respectively, and hydrogen bonds between AXL Ig1 and scFv are indicated with yellow dotted lines. Numbering of AXL Ig1 amino acids according to Supplementary Figure S3.

Based on the experimental alanine scanning data, the paratope residues that contributed most to binding – those that either abolished all binding or resulted in very weak binding with quick dissociation – are summarised in Table 1. Where other amino acids were tested at these locations, they were also not tolerated (Figure 5). To support the understanding of these essential interactions within the complex, we utilised AI-based prediction using AlphaFold3 (Figure 6A and B). The model and the specific molecular interactions that help stabilise the complex are shown in Figure 6. Contact residues of the V_H_ CDR loops can be seen buried within the interacting interface, participating in hydrogen bonds and van der Waals interactions with the AXL Ig1 domain.

**Table 1:** Key amino acids of tilvestamab contributing to binding of AXL Ig1 domain. AHo: the numbering of the amino acid according to the numbering scheme of Honegger and Plückthun [24]. scFv: numbering of the amino acid within the scFv-osmY fusion protein.

CDR	AHo	scFv	Residue
V_H_ CDR1	40	34	Y
V_H_ CDR1	42	36	N
V_H_ CDR2	57	51	R
V_H_ CDR3	109	100	R
V_H_ CDR3	134	104	Y
V_H_ CDR3	137	107	D
V_L_ CDR1	33	168	H
V_L_ CDR1	37	171	G
V_L_ CDR1	38	172	I
V_L_ CDR1	40	174	Y
V_L_ CDR3	108	232	Q
V_L_ CDR3	136	237	P

The hydroxyl group in the side chain of Tyr34 forms a hydrogen bond with the carboxylate group in the side chain of Asp62 of AXL Ig1 (numbering of AXL Ig1 according to Supplementary Figure S3). The guanidinium group, in the vicinity of Tyr34, of Arg51 forms hydrogen bonds with the backbone carbonyl group of Glu60 and the side chain carbonyl oxygen of Gln61. Moreover, in CDR3, Arg100 nearby forms two hydrogen bonds with the backbone and side chain carbonyl oxygens of Gln61, while the side chains of Tyr104 and Asp107 contribute to van der Waals interactions at the interface that would weaken upon mutation to Ala (Figure 6C).

Direct interactions of AXL Ig1 are less prevalent with residues of the V_L_ CDR loops. The nitrogen in the histidine imidazole group of His168 forms a hydrogen bond with the carboxyl group in Asp59 of AXL Ig1 (Figure 6D). Another hydrogen bond is formed between the hydroxyl group in the aromatic ring of Tyr174 and the side chain amide of Gln27, while the hydrophobic side chain of the Ile172 residue, being near the also hydrophobic Ile65 of AXL Ig1, can participate in van der Waals interactions. The remaining residues listed, Gly171, Gln232 and Pro237, do not tolerate mutations to Ala, as such changes could disrupt CDR loop flexibility and conformation.

Sites that tolerated mutation to alanine but showed variable tolerance for other substitutions are listed in Table 2. Specifically, when looking at the AlphaFold3 model of scFv-AXL Ig1, Phe33 has close interactions with AXL through its aromatic side chain. Mutations to Ala or Tyr at that se had no effect (Figure 5A), indicating a hydrophobic and/or aromatic residue at this position is important. The F33H and F33N mutants make the side chain – and the surface – more hydrophilic, while Pro at this site would affect the conformation of the entire CDR1 loop. Phe53 nearby also interacts with AXL via its side chain. Most mutations are tolerated, except for substitution to Pro and the acidic Asp; these substitutions could affect the local conformation and surface charge, respectively. Lys102 and Tyr103 in V_H_ CDR3 both make van der Waals contacts to AXL, and Tyr103 also makes a hydrogen bond to the carbonyl of Pro4 in AXL. In the experiment, most mutations in Lys102 and Tyr103 affected binding, which can be explained by the loss of these interactions. The most significant impact being the mutation to Asp, introducing an unfavourable negative charge into the binding site.

**Table 2:** Additional amino acids of tilvestamab contributing to binding of AXL Ig1 domain. The sites listed tolerate mutation to alanine, but not to the above substitutions. AHo: the numbering of the amino acid according to the numbering scheme of Honegger and Plückthun [24]. scFv: numbering of the amino acid within the scFv-oY fusion protein.

CDR	AHo	scFv	Residue	Substitutions
V_H_ CDR1	39	33	F	H, N, P
V_H_ CDR2	59	53	F	D, P
V_H_ CDR3	111	102	L	D, H, N, P
V_H_ CDR3	112	103	Y	D, H, N
V_L_ CDR2	57	191	Y	P
V_L_ CDR2	58	192	R	D, P
V_L_ CDR2	69	195	N	Y
V_L_ CDR3	109	233	G	D, H, P, Y
V_L_ CDR3	137	238	P	D, H, Y

**Figure 7:**
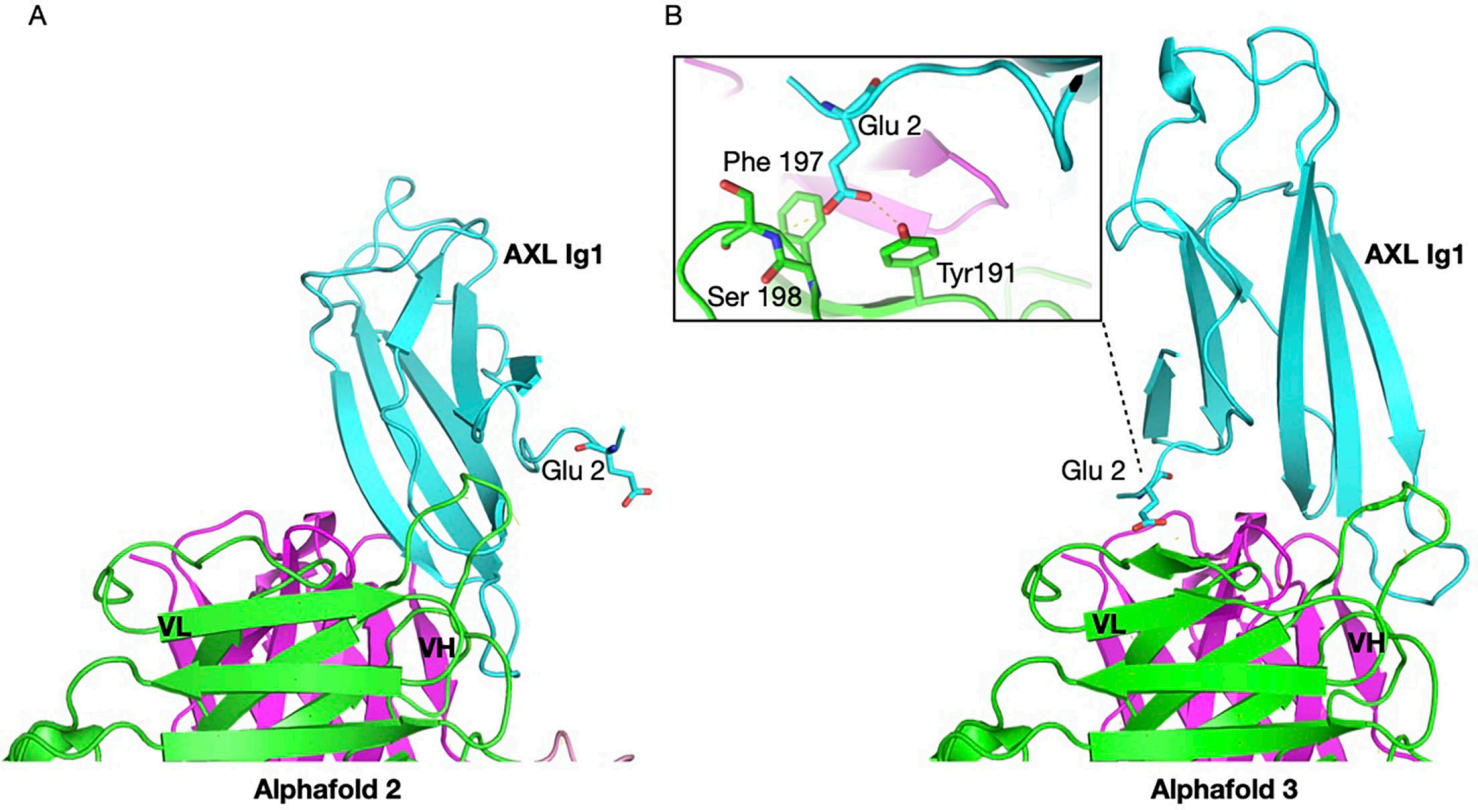
AlphaFold2 and AlphaFold3 comparison for predicting scFv-AXL Ig1 complex from their amino acid sequence. (**A**) AlphaFold2 structural model shows the N-terminus of AXL not interacting with scFv tilvestamab, whereas (**B**) AlphaFold3 provides a more accurate structure prediction, modelling Glu2 interacting with residues of tilvestamab. This prediction aligns with the experimental work. Numbering of AXL Ig1 amino acids according to Supplementary Figure S3. The protein colouring is consistent with Figure 6.

Tyr191, Arg192 and Asn195 in V_L_ CDR2 appear to form a structurally critical region for the complex. The most drastic effects arise when Tyr191 or Arg192 are mutated to Pro and Asp, indicating importance for the correct conformation of CDR2 and lack of tolerance for a negative charge at the paratope. Results in the supplementary material show that tilvestamab binds to the Ig1 domain of human AXL and rhesus monkey AXL, but not mouse AXL protein (Supplementary Figure S4), and a single glutamate, Glu2, close to the N terminus of the mature AXL protein, is essential for binding (Supplementary Figure S5). scFv-AXL Ig1 complex modelled by AlphaFold3 agrees with Glu2 of AXL being important for binding tilvestamab, as Tyr191 and Ser198 form hydrogen bonds with Glu2, and the hydrophobic Phe197 additionally stabilises the protein interface (Figure 7). AlphaFold2 models AXL Ig1 in a different orientation, with Glu2 not interacting with tilvestamab, whereas AlphaFold3 apparently improves the accuracy of the protein complex, aligning with the experimental observations and the importance of Glu2 (Figure 7).

Lastly, in the V_L_ CDR3 loop, Gly233 and Pro238 appear structurally critical, but neither makes direct contact with AXL. These are the most flexible and most rigid amino acids. With the hydrogen atom side chain of Gly233 pointing towards the V_H_ chain, substitutions with smaller amino acids S and A would be tolerated, but larger or charged residues such as D, H, P or Y would be more disruptive in that position, as reflected in the experimental binding data. In addition, the Gly233 carbonyl group forms a hydrogen bond with Gln61 of AXL, and the geometry of this bond would be affected by mutations. Mutations at Pro238, located at the end of the CDR3, affect the most rigid amino acid, and all mutations influence binding, likely due to changes in the conformation of the CDR3 loop.

**Figure 8:**
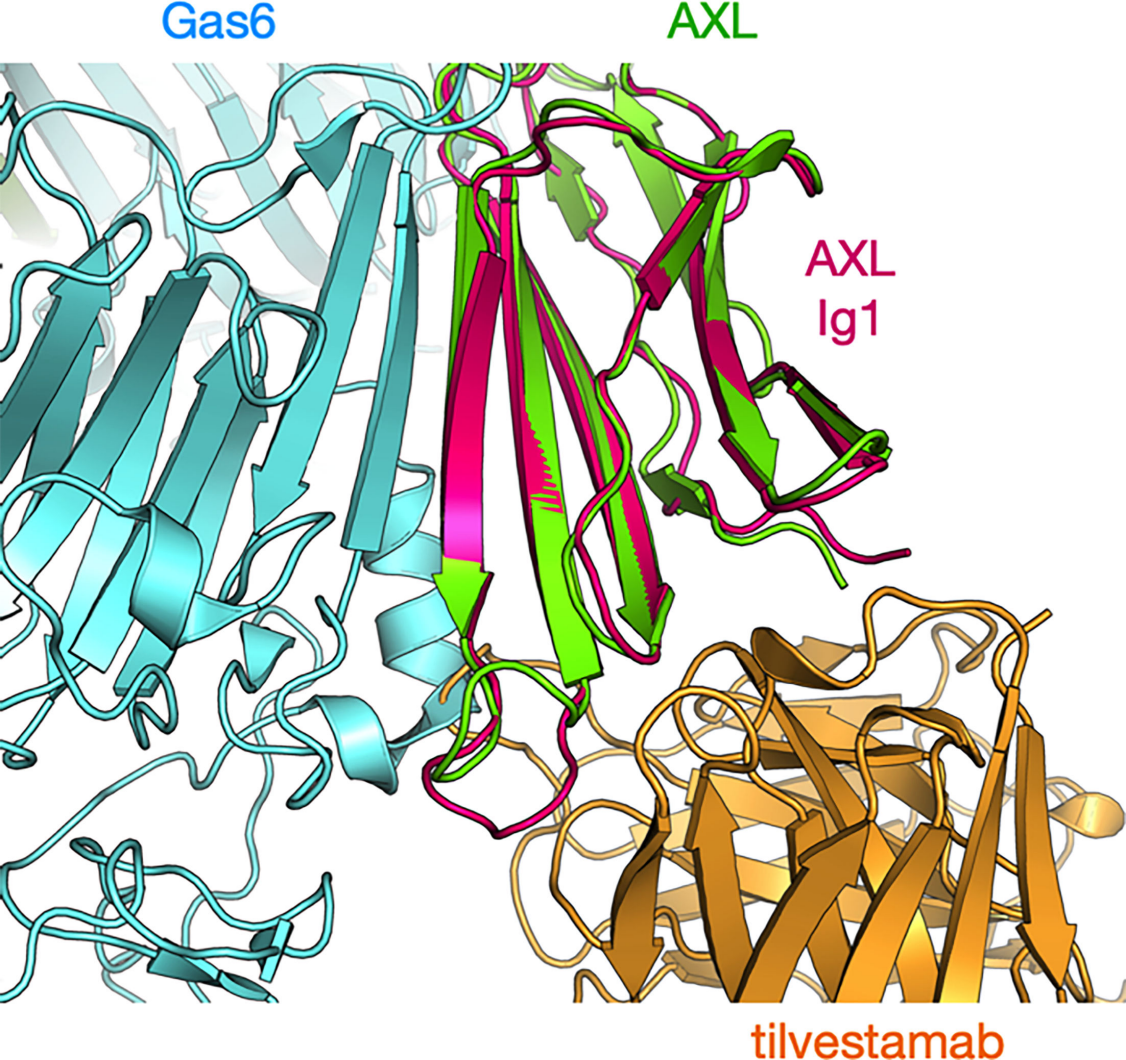
Superposition of the AlphaFold3 model (magenta/orange) and the AXL-GAS6 crystal structure (green/cyan). Note a small overlap in GAS6 and tilvestamab, which occupy binding surfaces close to each other on AXL Ig1.

Tilvestamab is known to block AXL signalling [25,26], while it reduced GAS6 binding to AXL only by ~50% [16]. This suggests that tilvestamab does not simply block GAS6 binding to AXL but affects the formation of a signalling-competent AXL dimer upon GAS6 interaction. To get an insight into this subject, we superposed the AlphaFold3 prediction of the AXL Ig1 – tilvestamab scFv complex on the crystal structure of the complex between AXL and GAS6 (PDB entry 2c5d) [27]. The superposition (Figure 8) shows that the binding sites for tilvestamab and GAS6 lie close on the surface of AXL Ig1, with a small overlap. The N terminus of AXL, with Glu2, does not interact with GAS6 in the crystal structure. These observations suggest that tilvestamab may reduce the binding affinity of GAS6 and affect the conformation and oligomeric state of the protein complex, such that a signalling-competent AXL receptor dimer cannot form.

## Conclusions

We have carried out an experimental screening of the CDR loops of tilvestamab to pinpoint key residues for AXL binding, and the AlphaFold3 model of the tilvestamab-AXL complex is in line with the mutagenesis data. Several interaction hotspots were identified, and one of the most interesting concerns the interactions of AXL residue Glu2, which is crucial for the interaction. Of note, our ongoing follow-up work for obtaining an experimental 3D structure for the AXL-tilvestamab complex has indicated that while AlphaFold3 identified the binding interfaces correctly here, the orientation of the interacting domains in the cryo-EM structure is different [28]. However, the latter still fits our paratope screening data, indicating the scFv approach described here can provide functional information while using bacterial culture supernatants for screening. Overall, our data point towards possibilities of affecting tilvestamab affinity further for various applications.

## Supplementary material

Online supplementary material 1

## Data Availability

All relevant data are included within the manuscript figures, tables, and supplementary information. Raw data files are available from the corresponding author upon reasonable request.

## Abbreviations

BLI: biolayer interferometry
CDR: complementarity-determining region
GAS6: growth arrest-specific protein 6
TAM: TYRO3, AXL and MERTK
TBS-T: Tris-buffered saline/Tween-20
V_H_: heavy chain
V_L_: light chain
mAb: monoclonal antibody
osmY: osmotically inducible protein Y
scFv: single-chain variable fragment
