# Supplementary material for: Paratope mapping of tilvestamab, an anti-AXL function-blocking antibody, using high-throughput bacterial expression of secreted scFv-osmY fusion proteins
Source: Biosci Rep. 2025 Oct 28;45(10):635–47. doi: 10.1042/BSR20253747 (PMC12784346; doi:10.1042/BSR20253747)
Supplement: Online supplementary material 1 [file bsr-45-10-BSR20253747-s001.pdf]

Supplementary Material for

Paratope mapping of tilvestamab, an anti-AXL function blocking antibody, using high-throughput bacterial expression of secreted scFv-ompY fusion proteins

Eleni Christakou, Petri Kursula, David Micklem

Corresponding author: Petri Kursula, petri.kursula@uib.no

This PDF file includes:

Supplemental Methods

Supplemental Figures 1-4

Supplemental Tables 1-2

Supplementary Methods

Surface plasmon resonance analysis of binding of antibodies to AXL from different species

Recombinant proteins in **Supplementary Figure 4A:** human AXL-Fc chimera (Evitria), recombinant mouse Axl-Fc chimera (R&D Systems 854-AX), and recombinant rhesus monkey AXL-Fc chimera were coupled to a CM5 sensor chip (Biacore BR-1000-14) using an amine coupling kit (Biacore BR-1000-50). Briefly, proteins were adjusted to 5 µg/ml in 10 mM sodium acetate pH 4.8 and the immobilisation protocol was run on a Biacore 3000 (GE Healthcare), aiming for 100 RU coupling. Chip performance was tested using antibody YW327.6S2var (Evitria) at 10 µg/ml in HBS-EP (Biacore 1001-88) with 1 min injection time and 20 µl/min flow rate for 10 cycles using 10 mM HCl and 1M NaCl as regeneration solution (30 s at 50µl/min followed by 1 min stabilisation). Binding of tilvestamab was tested similarly using 7.5 µg/ml antibody.

Biolayer interferometry analysis of binding of antibodies to AXL variants

Recombinant proteins in **Supplementary Figure 4C** were captured on streptavidin-coated dip and read biosensors (Forte-Bio 18-5019) by pre-treating the tips with 20 µg/ml biotin-conjugated donkey anti-human IgG Fc (Jackson ImmunoResearch 709-065-098) in PBS for 60 s. Tips were then saturated with antibody at 20 µg/ml in PBS for 300 s. A baseline was taken (60 s PBS) before a tip was immersed in each of the recombinant antigens and the association measured for 600 s. Tips were regenerated by three cycles of immersion in 10 mM glycine, pH 1.5 for 5 s followed by neutralisation in PBS for 5 s, before continuing with the next antibody. Antibodies were tested in the order tilvestamab then YW327.6S2var. For association with 1H12, the order of loading and association was reversed, as the mouse antibody 1H12 does not bind to the donkey anti-human IgG Fc. Recombinant proteins in **Supplementary Figure 5B** were dialysed

to PBS and biotinylated with EZ-Link NHS-PEG4-Biotin (ThermoFisher 21329) by reconstituting the reagent at 20 mM in DMSO and diluting to 0.2 mM with water. 5 µl diluted biotinylation reagent was added to 1 ml recombinant protein, incubated for 30 min and dialysed extensively against PBS. Biotinylated recombinant proteins were captured on streptavidin-coated dip and read biosensors for 1200 s, a 600 s baseline was recorded before association with tilvestamab (10 µg/ml) or YW327.6S2var (6.6 µg/ml) for 100 s and dissociation in buffer for 600 s using fresh biosensors for each antibody.

Production of recombinant proteins for epitope mapping

Recombinant proteins were purchased from Evitria (Switzerland) or produced in-house by cloning the desired coding sequence into plasmid pCMV-3Tag-1A (Agilent). In each case, protein was produced by transient transfection of CHO cells followed by purification of the Fc-fusion proteins over a Protein A column (MAbSelect XTRA, GE Healthcare) with elution at low pH.

Sequences of recombinant proteins and plasmids

For proteins produced commercially, the final sequence is given, as the signal peptides used are proprietary. For proteins produced in-house, signal peptides are given in lower-case.

Human AXL Fc (AXL Fc, Evitria)

```
EESPFVGNPGNITGARGLTGTLRCQLQVQGEPPEVHWLRDGQILELADSTQTQVPLGEDEQDDWIVVSQLRITSL
QLSDTGQYQCLVFLGHQTFVSQPGYVGLEGLPYFLEEPEDRTVAANTPFNLSCQAQGPPEPVDLLWLQDAVPLAT
APGHGPQRSLHVPGLNKTSSFSCEAHNAKGVTTSRTATITVLPQQPRNLHLVSRQPTELEVAWTPGLSGIYPLTH
CTLQAVLSDDGMGIQAGEPDPPEEPLTSQASVPPHQLRLGSLHPHTPYHIRVACTSSQGPSSWTHWLPVETPEGV
PLGPPENISATRNGSQAFVHWQEPRAPLQGTLLGYRLAYQGQDTPEVLMDIGLRQEVTLELQGDGSVSNLTVCVA
AYTAAGDGPWSLPVPLEAWRPGQAQPVHQLVKEGGGGSGGGGSGGGGSDKTHTCPPCPAPELLGGPSVFLFPPKP
KDTLMISRTPEVTCVVVDVSHEDPEVKFNWYVDGVEVHNAKTKPREEQYNSTYRVVSVLTVLHQDWLNGKEYKCK
VSNKALPAPIEKTISKAKGQPREPQVYTLPPSREEMTKNQVSLTCLVKGFYPSDIAVEWESNGQPENNYKTTPPV
LDSDGSFFLYSKLTVDKSRWQQGNVFSCSVMHEALHNHYTQKSLSLSPGK
```

Rhesus Monkey AXL-Fc (RheAXL Fc, Evitria)

```
EESPFVGNPGNITGARGLTGTLRCQLQVQGEPPEVHWLRDGQILELADSTQTQVPLGEDEQDDWIVVSQLRIASL
QLSDAGQYQCLVFLGHQNFVSQPGYVGLEGLPYFLEEPEDRTVAANTHFNLSCQAQGPPEPVDLLWLQDAVPLAT
```

APGHGPQRNLHVPGLNKTSSFSCEAHNAKGVTTSRTATITVLPQQPRNLHLVSRQPTELEVAWTPGLSGIYPLTH
CTLQAMLSDNEVGIQAGEPDPPEEPLTLQASVPPHQLRLGSLHPHTPYHIRVACTSSQGPSSWTHWLPVETPEGV
PLGPPENISATRNGSQAFVHWQEPRAPLQGTLLGYRLAYQGQDTPEVLMDIGLRQEVTLELQGDGSVSNLTVCVA
AYTAAGDGPWSLPVPLEAWRPGQAQPVHQLVKEGGGGSGGGGSGGGGSDKTHTCPPCPAPELLGGPSVFLFPPKP
KDTLMISRTPEVTCVVVDVSHEDPEVKFNWYVDGVEVHNAKTKPREEQYNSTYRVVSVLTVLHQDWLNGKEYKCK
VSNKALPAPIEKTISKAKGQPREPQVYTLPPSREEMTKNQVSLTCLVKGFYPSDIAVEWESNGQPENNYKTTPPV
LDSDGSFFLYSKLTVDKSRWQQGNVFSCSVMHEALHNHYTQKSLSLSPGK

Mouse AXL-Fc (MmAXL Fc, RnD Systems)

HKDTQTEAGSPFVGNPGNITGARGLTGTLRCELQVQGEPPEVVWLRDGQILELADNTQTQVPLGEDWQDEWKVVS
QLRISALQLSDAGEYQCMVHLEGRTFVSQPGFVGLEGLPYFLEEPEDKAVPANTPFNLSCQAQGPPEPVTLLWLQ
DAVPLAPVTGHSSQHSLQTPGLNKTSSFSCEAHNAKGVTTSRTATITVLPQRPHHLHVVSRQPTELEVAWTPGLS
GIYPLTHCNLQAVLSDDGVGIWLGKSDPPEDPLTLQVSVPPHQLRLEKLLPHTPYHIRISCSSSQGPSPWTHWLP
VETTEGVPLGPPENVSAMRNGSQVLVRWQEPRVPLQGTLLGYRLAYRGQDTPEVLMDIGLTREVTLELRGDRPVA
NLTVSVTAYTSAGDGPWSLPVPLEPWRPGQGQPLHHLVSEPPPRAFSWPIEGRMDPEPRGPTIKPCPPCKCPAPN
LLGGPSVFIFPPKIKDVLMISLSPIVTCVVVDVSEDDPDVQISWFVNNVEVHTAQTQTHREDYNSTLRVVSALPI
QHQDWMSGKEFKCKVNNKDLPAPIERTISKPKGSVRAPQVYVLPPPEEEMTKKQVTLTCMVTDFMPEDIYVEWTN
NGKTELNYKNTEPVLDSDGSYFMYSKLRVEKKNWVERNSYSCSVVHEGLHNHHTTKSFSRTPGK

AXL IG1-Fc

mawrcprmgrvplawclalcgwacmaprgtqaEESPFVGNPGNITGARGLTGTLRCQLQVQGEPPEVHWLRDGQI
LELADSTQTQVPLGEDEQDDWIVVSQLRITSLQLSDTGQYQCLVFLGHQTFVSQPGYVGLEGSGGGGSGGGGSGG
GGSDKTHTCPPCPAPELLGGPSVFLFPPKPKDTLMISRTPEVTCVVVDVSHEDPEVKFNWYVDGVEVHNAKTKPR
EEQYNSTYRVVSVLTVLHQDWLNGKEYKCKVSNKALPAPIEKTISKAKGQPREPQVYTLPPSRDELTKNQVSLTC
LVKGFYPSDIAVEWESNGQPENNYKTTPPVLDSDGSFFLYSKLTVDKSRWQQGNVFSCSVMHEALHNHYTQKSLS
LSPGK

MsHsAxl-Fc

mgrvplawwlalccwgcaaHKDTQTEAGSPFVGNPGNITGARGLTGTLRCQLQVQGEPPEVHWLRDGQILELADS
TQTQVPLGEDEQDDWIVVSQLRITSLQLSDTGQYQCLVFLGHQTFVSQPGYVGLEGLPYFLEEPEDKAVPANTPF
NLSCQAQGPPEPVTLLWLQDAVPLAPVTGHSSQHSLQTPGLNKTSSFSCEAHNAKGVTTSRTATITVLPQRPHHL
HVVSRQPTELEVAWTPGLSGIYPLTHCNLQAVLSDDGVGIWLGKSDPPEDPLTLQVSVPPHQLRLEKLLPHTPYH
IRISCSSSQGPSPWTHWLPVETTEGVPLGPPENVSAMRNGSQVLVRWQEPRVPLQGTLLGYRLAYRGQDTPEVLM
DIGLTREVTLELRGDRPVANLTVSVTAYTSAGDGPWSLPVPLEPWRPGQGQPLHHLVSEPISGGGGSGGGGSGGG
GSDKTHTCPPCPAPELLGGPSVFLFPPKPKDTLMISRTPEVTCVVVDVSHEDPEVKFNWYVDGVEVHNAKTKPRE
EQYNSTYRVVSVLTVLHQDWLNGKEYKCKVSNKALPAPIEKTISKAKGQPREPQVYTLPPSRDELTKNQVSLTCL
VKGFYPSDIAVEWESNGQPENNYKTTPPVLDSDGSFFLYSKLTVDKSRWQQGNVFSCSVMHEALHNHYTQKSLSL
SPGK

MsHsAxl-Fc EAG>EE

mgrvplawwlalccwgcaaHKDTQTEESPFVGNPGNITGARGLTGTLRCQLQVQGEPPEVHWLRDGQILELADST
QTQVPLGEDEQDDWIVVSQLRITSLQLSDTGQYQCLVFLGHQTFVSQPGYVGLEGLPYFLEEPEDKAVPANTPFN
LSCQAQGPPEPVTLLWLQDAVPLAPVTGHSSQHSLQTPGLNKTSSFSCEAHNAKGVTTSRTATITVLPQRPHHLH
VVSRQPTELEVAWTPGLSGIYPLTHCNLQAVLSDDGVGIWLGKSDPPEDPLTLQVSVPPHQLRLEKLLPHTPYHI
RISCSSSQGPSPWTHWLPVETTEGVPLGPPENVSAMRNGSQVLVRWQEPRVPLQGTLLGYRLAYRGQDTPEVLMD
IGLTREVTLELRGDRPVANLTVSVTAYTSAGDGPWSLPVPLEPWRPGQGQPLHHLVSEPISGGGGSGGGGSGGGG
SDKTHTCPPCPAPELLGGPSVFLFPPKPKDTLMISRTPEVTCVVVDVSHEDPEVKFNWYVDGVEVHNAKTKPREE
QYNSTYRVVSVLTVLHQDWLNGKEYKCKVSNKALPAPIEKTISKAKGQPREPQVYTLPPSRDELTKNQVSLTCLV
KGFYPSDIAVEWESNGQPENNYKTTPPVLDSDGSFFLYSKLTVDKSRWQQGNVFSCSVMHEALHNHYTQKSLSLS
PGK

Rat AXL-Fc (RnAXL Fc, Evitria)

```
EADSPFVGNPGNITGARGLTGTLRCELQVQGEPPEVMWLRDGQILELADNTQTQVPLGEDWQDEWKVVSQLRISA
LQLSDAGEYQCMVHLEGRTFVSQPGFVGLEGLPYFLEEPEDKAVPANTPFNLSCQAQGPPEPVTLLWLQDAVPLA
PVAGYSFQHSLQAPGLNKTSSFSCEAHNAKGVTTSRTATITVLPQRPHNLHVVSRHPTELEVAWIPTLSGIYPLT
HCTLQAVLSNDGVGVWLGKSDPPEEPLTVQVSVPPHQLRLEKLLPHTPYHIRVSCTSSQGPSPWTHWLPVETTEG
VPLGPPENVSAMRNGSQALVRWQEPREPLQGTLLGYRLAYRGQDTPEVLMDIGLTREVTLELRGDRPVANLTVSV
AAYTSAGDGPWSLPVPLEPWRPGQGQPLHHLVSEGGGGSDKTHTCPPCPAPELLGGPSVELFPPKPKDTLMISRT
PEVTCVVVDVSHEDPEVKFNWYVDGVEVHNAKTKPREEQYNSTYRVVSVLTVLHQDWLNGKEYKCKVSNKALPAP
IEKTISKAKGQPREPQVYTLPPSREEMTKNQVSLTCLVKGFYPSDIAVEWESNGQPENNYKTTPPVLDSDGSFFL
YSKLTVDKSRWOOGNVFSCSVMHEALHNHYTOKSLSLSPGK
```

BGB289 pET-22b (+) (see **Supplementary Figure 1)**

```
FEATURES             Location/Qualifiers
     rep_origin      10..465
                     /note="f1 origin"
     CDS             597..1454
                     /note="bla AmpR"
     rep_origin      2215
                     /note="ColE1 pBR322 origin"
     CDS             complement(2646..2837)
                     /note="Rop"
     CDS             complement(3649..4728)
                     /note="lacI"
     regulatory      5115..5131
                     /regulatory_class="promoter"
                     /note=""
                     /note="T7 promoter"
     regulatory      5134..5158
                     /regulatory_class=""
                     /note="lac operator"
     CDS             5203..5286
                     /note="osmY signal peptide"
     misc_feature    5287..>5294
                     /note="SfiI/RI multiple cloning site"
     CDS             5299..6081
                     /note="Recombinant antibody fragment;"
     CDS             5302..5655
                     /note="H2L1 VH domain"
     CDS             5710..6045
                     /note="H2L1 VL domain"
     CDS             6082..6600
                     /locus_tag="SR36_22250"
                     /note="osmY"
                     /product="periplasmic protein"
     CDS             6601..6666
                     /note="Myc-His Tag"
     regulatory      6731..6777
                     /regulatory_class="terminator"
                     /note=""
                     /note="T7 terminator"
ORIGIN
        1 GCGAATGGGA CGCGCCCTGT AGCGGCGCAT TAAGCGCGGC GGGTGTGGTG GTTACGCGCA
       61 GCGTGACCGC TACACTTGCC AGCGCCCTAG CGCCCGCTCC TTTCGCTTTC TTCCCTTCCT
      121 TTCTCGCCAC GTTCGCCGGC TTTCCCCGTC AAGCTCTAAA TCGGGGGCTC CCTTTAGGGT
      181 TCCGATTTAG TGCTTTACGG CACCTCGACC CCAAAAAACT TGATTAGGGT GATGGTTCAC
      241 GTAGTGGGCC ATCGCCCTGA TAGACGGTTT TTCGCCCTTT GACGTTGGAG TCCACGTTCT
      301 TTAATAGTGG ACTCTTGTTC CAAACTGGAA CAACACTCAA CCCTATCTCG GTCTATTCTT
      361 TTGATTTATA AGGGATTTTG CCGATTTCGG CCTATTGGTT AAAAAATGAG CTGATTTAAC
      421 AAAAATTTAA CGCGAATTTT AACAAAATAT TAACGTTTAC AATTTCAGGT GGCACTTTTC
      481 GGGGAAATGT GCGCGGAACC CCTATTTGTT TATTTTTCTA AATACATTCA AATATGTATC
```

541 CGCTCATGAG ACAATAACCC TGATAAATGC TTCAATAATA TTGAAAAAGG AAGAGTATGA
601 GTATTCAACA TTTCCGTGTC GCCCTTATTC CCTTTTTTGC GGCATTTTGC CTTCCTGTTT
661 TTGCTCACCC AGAAACGCTG GTGAAAGTAA AAGATGCTGA AGATCAGTTG GGTGCACGAG
721 TGGGTTACAT CGAACTGGAT CTCAACAGCG GTAAGATCCT TGAGAGTTTT CGCCCCGAAG
781 AACGTTTTCC AATGATGAGC ACTTTTAAAG TTCTGCTATG TGGCGCGGTA TTATCCCGTA
841 TTGACGCCGG GCAAGAGCAA CTCGGTCGCC GCATACACTA TTCTCAGAAT GACTTGGTTG
901 AGTACTCACC AGTCACAGAA AAGCATCTTA CGGATGGCAT GACAGTAAGA GAATTATGCA
961 GTGCTGCCAT AACCATGAGT GATAACACTG CGGCCAACTT ACTTCTGACA ACGATCGGAG
1021 GACCGAAGGA GCTAACCGCT TTTTTGCACA ACATGGGGGA TCATGTAACT CGCCTTGATC
1081 GTTGGGAACC GGAGCTGAAT GAAGCCATAC CAAACGACGA GCGTGACACC ACGATGCCTG
1141 CAGCAATGGC AACAACGTTG CGCAAACTAT TAACTGGCGA ACTACTTACT CTAGCTTCCC
1201 GGCAACAATT AATAGACTGG ATGGAGGCGG ATAAAGTTGC AGGACCACTT CTGCGCTCGG
1261 CCCTTCCGGC TGGCTGGTTT ATTGCTGATA AATCTGGAGC CGGTGAGCGT GGGTCTCGCG
1321 GTATCATTGC AGCACTGGGG CCAGATGGTA AGCCCTCCCG TATCGTAGTT ATCTACACGA
1381 CGGGGAGTCA GGCAACTATG GATGAACGAA ATAGACAGAT CGCTGAGATA GGTGCCTCAC
1441 TGATTAAGCA TTGGTAACTG TCAGACCAAG TTTACTCATA TATACTTTAG ATTGATTTAA
1501 AACTTCATTT TTAATTTAAA AGGATCTAGG TGAAGATCCT TTTTGATAAT CTCATGACCA
1561 AAATCCCTTA ACGTGAGTTT TCGTTCCACT GAGCGTCAGA CCCCGTAGAA AAGATCAAAG
1621 GATCTTCTTG AGATCCTTTT TTTCTGCGCG TAATCTGCTG CTTGCAAACA AAAAAACCAC
1681 CGCTACCAGC GGTGGTTTGT TTGCCGGATC AAGAGCTACC AACTCTTTTT CCGAAGGTAA
1741 CTGGCTTCAG CAGAGCGCAG ATACCAAATA CTGTCCTTCT AGTGTAGCCG TAGTTAGGCC
1801 ACCACTTCAA GAACTCTGTA GCACCGCCTA CATACCTCGC TCTGCTAATC CTGTTACCAG
1861 TGGCTGCTGC CAGTGGCGAT AAGTCGTGTC TTACCGGGTT GGACTCAAGA CGATAGTTAC
1921 CGGATAAGGC GCAGCGGTCG GGCTGAACGG GGGGTTCGTG CACACAGCCC AGCTTGGAGC
1981 GAACGACCTA CACCGAACTG AGATACCTAC AGCGTGAGCT ATGAGAAAGC GCCACGCTTC
2041 CCGAAGGGAG AAAGGCGGAC AGGTATCCGG TAAGCGGCAG GGTCGGAACA GGAGAGCGCA
2101 CGAGGGAGCT TCCAGGGGGA AACGCCTGGT ATCTTTATAG TCCTGTCGGG TTTCGCCACC
2161 TCTGACTTGA GCGTCGATTT TTGTGATGCT CGTCAGGGGG GCGGAGCCTA TGGAAAAACG
2221 CCAGCAACGC GGCCTTTTTA CGGTTCCTGG CCTTTTGCTG GCCTTTTGCT CACATGTTCT
2281 TTCCTGCGTT ATCCCCTGAT TCTGTGGATA ACCGTATTAC CGCCTTTGAG TGAGCTGATA
2341 CCGCTCGCCG CAGCCGAACG ACCGAGCGCA GCGAGTCAGT GAGCGAGGAA GCGGAAGAGC
2401 GCCTGATGCG GTATTTTCTC CTTACGCATC TGTGCGGTAT TTCACACCGC ATATATGGTG
2461 CACTCTCAGT ACAATCTGCT CTGATGCCGC ATAGTTAAGC CAGTATACAC TCCGCTATCG
2521 CTACGTGACT GGGTCATGGC TGCGCCCCGA CACCCGCCAA CACCCGCTGA CGCGCCCTGA
2581 CGGGCTTGTC TGCTCCCGGC ATCCGCTTAC AGACAAGCTG TGACCGTCTC CGGGAGCTGC
2641 ATGTGTCAGA GGTTTTCACC GTCATCACCG AAACGCGCGA GGCAGCTGCG GTAAAGCTCA
2701 TCAGCGTGGT CGTGAAGCGA TTCACAGATG TCTGCCTGTT CATCCGCGTC CAGCTCGTTG
2761 AGTTTCTCCA GAAGCGTTAA TGTCTGGCTT CTGATAAAGC GGGCCATGTT AAGGGCGGTT
2821 TTTTCCTGTT TGGTCACTGA TGCCTCCGTG TAAGGGGGAT TTCTGTTCAT GGGGGTAATG
2881 ATACCGATGA AACGAGAGAG GATGCTCACG ATACGGGTTA CTGATGATGA ACATGCCCGG
2941 TTACTGGAAC GTTGTGAGGG TAAACAACTG GCGGTATGGA TGCGGCGGGA CCAGAGAAAA
3001 ATCACTCAGG GTCAATGCCA GCGCTTCGTT AATACAGATG TAGGTGTTCC ACAGGGTAGC
3061 CAGCAGCATC CTGCGATGCA GATCCGGAAC ATAATGGTGC AGGGCGCTGA CTTCCGCGTT
3121 TCCAGACTTT ACGAAACACG GAAACCGAAG ACCATTCATG TTGTTGCTCA GGTCGCAGAC
3181 GTTTTGCAGC AGCAGTCGCT TCACGTTCGC TCGCGTATCG GTGATTCATT CTGCTAACCA
3241 GTAAGGCAAC CCCGCCAGCC TAGCCGGGTC CTCAACGACA GGAGCACGAT CATGCGCACC
3301 CGTGGGGCCG CCATGCCGGC GATAATGGCC TGCTTCTCGC CGAAACGTTT GGTGGCGGGA
3361 CCAGTGACGA AGGCTTGAGC GAGGGCGTGC AAGATTCCGA ATACCGCAAG CGACAGGCCG
3421 ATCATCGTCG CGCTCCAGCG AAAGCGGTCC TCGCCGAAAA TGACCCAGAG CGCTGCCGGC
3481 ACCTGTCCTA CGAGTTGCAT GATAAAGAAG ACAGTCATAA GTGCGGCGAC GATAGTCATG
3541 CCCCGCGCCC ACCGGAAGGA GCTGACTGGG TTGAAGGCTC TCAAGGGCAT CGGTCGAGAT
3601 CCCGGTGCCT AATGAGTGAG CTAACTTACA TTAATTGCGT TGCGCTCACT GCCCGCTTTC
3661 CAGTCGGGAA ACCTGTCGTG CCAGCTGCAT TAATGAATCG GCCAACGCGC GGGGAGAGGC
3721 GGTTTGCGTA TTGGGCGCCA GGGTGGTTTT TCTTTTCACC AGTGAGACGG GCAACAGCTG
3781 ATTGCCCTTC ACCGCCTGGC CCTGAGAGAG TTGCAGCAAG CGGTCCACGC TGGTTTGCCC
3841 CAGCAGGCGA AAATCCTGTT TGATGGTGGT TAACGGCGGG ATATAACATG AGCTGTCTTC
3901 GGTATCGTCG TATCCCACTA CCGAGATATC CGCACCAACG CGCAGCCCGG ACTCGGTAAT
3961 GGCGCGCATT GCGCCCAGCG CCATCTGATC GTTGGCAACC AGCATCGCAG TGGGAACGAT
4021 GCCCTCATTC AGCATTTGCA TGGTTTGTTG AAAACCGGAC ATGGCACTCC AGTCGCCTTC
4081 CCGTTCCGCT ATCGGCTGAA TTTGATTGCG AGTGAGATAT TTATGCCAGC CAGCCAGACG
4141 CAGACGCGCC GAGACAGAAC TTAATGGGCC CGCTAACAGC GCGATTTGCT GGTGACCCAA
4201 TGCGACCAGA TGCTCCACGC CCAGTCGCGT ACCGTCTTCA TGGGAGAAAA TAATACTGTT
4261 GATGGGTGTC TGGTCAGAGA CATCAAGAAA TAACGCCGGA ACATTAGTGC AGGCAGCTTC
4321 CACAGCAATG GCATCCTGGT CATCCAGCGG ATAGTTAATG ATCAGCCCAC TGACGCGTTG
4381 CGCGAGAAGA TTGTGCACCG CCGCTTTACA GGCTTCGACG CCGCTTCGTT CTACCATCGA
4441 CACCACCACG CTGGCACCCA GTTGATCGGC GCGAGATTTA ATCGCCGCGA CAATTTGCGA

4501 CGGCGCGTGC AGGGCCAGAC TGGAGGTGGC AACGCCAATC AGCAACGACT GTTTGCCCGC
4561 CAGTTGTTGT GCCACGCGGT TGGGAATGTA ATTCAGCTCC GCCATCGCCG CTTCCACTTT
4621 TTCCCGCGTT TTCGCAGAAA CGTGGCTGGC CTGGTTCACC ACGCGGGAAA CGGTCTGATA
4681 AGAGACACCG GCATACTCTG CGACATCGTA TAACGTTACT GGTTTCACAT TCACCACCCT
4741 GAATTGACTC TCTTCCGGGC GCTATCATGC CATACCGCGA AAGGTTTTGC GCCATTCGAT
4801 GGTGTCCGGG ATCTCGACGC TCTCCCTTAT GCGACTCCTG CATTAGGAAG CAGCCCAGTA
4861 GTAGGTTGAG GCCGTTGAGC ACCGCCGCCG CAAGGAATGG TGCATGCAAG GAGATGGCGC
4921 CCAACAGTCC CCCGGCCACG GGGCCTGCCA CCATACCCAC GCCGAAACAA GCGCTCATGA
4981 GCCCGAAGTG GCGAGCCCGA TCTTCCCCAT CGGTGATGTC GGCGATATAG GCGCCAGCAA
5041 CCGCACCTGT GGCGCCGGTG ATGCCGGCCA CGATGCGTCC GGCGTAGAGG ATCGAGATCT
5101 CGATCCCGCG AAATTAATAC GACTCACTAT AGGGGAATTG TGAGCGGATA ACAATTCCCC
5161 TCTAGAAATA ATTTTGTTTA ACTTTAAGAA GGAGATATAC ATATGACcAT GACccGtGTG
5221 AAGATTagcA AgACcCTGCT GGCgGTtATG cTGACCagcG CgGTgGCGAC CGGtagcGCg
5281 TAtGCGGGCC CAGCCGGCCT GGAAGTGCAG CTGGTTGAAA GCGGTGGCGG TCTGGTGCAA
5341 CCGGGCGGTA GCCTGCGTCT GAGCTGCGCG GCGAGCGGTT ACAGCTTCAC CGACTTTTAT
5401 ATCAACTGGG TGCGTCAGGC GCCGGGTAAA GGTCTGGAGT GGGTTGCGCG TATTTTCCCG
5461 GGCGGTGACA ACACCTACTA TAACGAAAAG TTCAAAGGTC GTTTTACCCT GAGCGCGGAT
5521 ACCAGCAAGA GCACCGCGTA CCTGCAGATG AACAGCCTGC GTGCGGAGGA CACCGCGGTT
5581 TACTATTGCG CGCGTCGTGG CCTGTACTAT GCGATGGATT ATTGGGGCCA AGGTACCCTG
5641 GTGACCGTTA GCAGCGCGAA AACCACCCCT CCTAAGCTTG AGGAAGGTGA ATTCAGCGAG
5701 GCACGCGTAG ACATCCAGAT GACCCAGAGC CCGAGCAGCC TGAGCGCGAG CGTGGGCGAT
5761 CGTGTTACCA TCACCTGCCG TAGCAGCCAG AGCCTGGTTC ACAGCAACGG TATTCCGTAC
5821 CTGCACTGGT ATCAGCAAAA GCCGGGCAAA GCGCCGAAGC TGCTGATCTA CCGTGTGAGC
5881 AACCGTTTCA GCGGTGTTCC GAGCCGTTTT AGCGGTAGCG GTAGCGGTAC CGACTTCACC
5941 CTGACCATTA GCAGCCTGCA ACCGGAGGAT TTTGCGACCT ACTATTGCAG CCAGGGTACC
6001 CATGTGCCGC CGACCTTCGG TCAAGGCACC AAAGTTGAAA TCAAGCGTGC GGATGCGGCG
6061 CCGACCGTGT CTGCGGCCGC TGAGAACAAC GCGCAGACCA CCAACGAAAG CGCGGGTCAA
6121 AAAGTGGATA GCAGCATGAA CAAGGTTGGC AACTTCATGG ACGATAGCGC GATTACCGCG
6181 AAGGTGAAAG CGGCGCTGGT TGACCACGAT AACATCAAAA GCACCGACAT TAGCGTGAAA
6241 ACCGATCAGA AGGTGGTTAC CCTGAGCGGT TTTGTTGAGA GCCAGGCGCA AGCGGAGGAA
6301 GCGGTGAAAG TTGCGAAGGG TGTGGAAGGC GTTACCAGCG TGAGCGACAA ACTGCACGTG
6361 CGTGATGCGA AAGAGGGTAG CGTTAAAGGT TATGCGGGTG ACACCGCGAC CACCAGCGAA
6421 ATCAAGGCGA AACTGCTGGC GGACGATATT GTGCCGAGCC GTCACGTGAA GGTTGAAACC
6481 ACCGACGGTG TGGTTCAACT GAGCGGCACC GTTGACAGCC AGGCGCAAAG CGATCGTGCG
6541 GAAAGCATTG CGAAAGCGGT TGATGGTGTG AAGAGCGTTA AAAACGACCT GAAAACCAAG
6601 GGATCCGAAC AGAAACTGAT TAGCGAAGAG GACCTGAGCC TCGAGCACCA CCACCACCAC
6661 CACTGAGATC CGGCTGCTAA CAAAGCCCGA AAGGAAGCTG AGTTGGCTGC TGCCACCGCT
6721 GAGCAATAAC TAGCATAACC CCTTGGGGCC TCTAAACGGG TCTTGAGGGG TTTTTTGCTG
6781 AAAGGAGGAA CTATATCCGG ATtg

Supplementary Figures

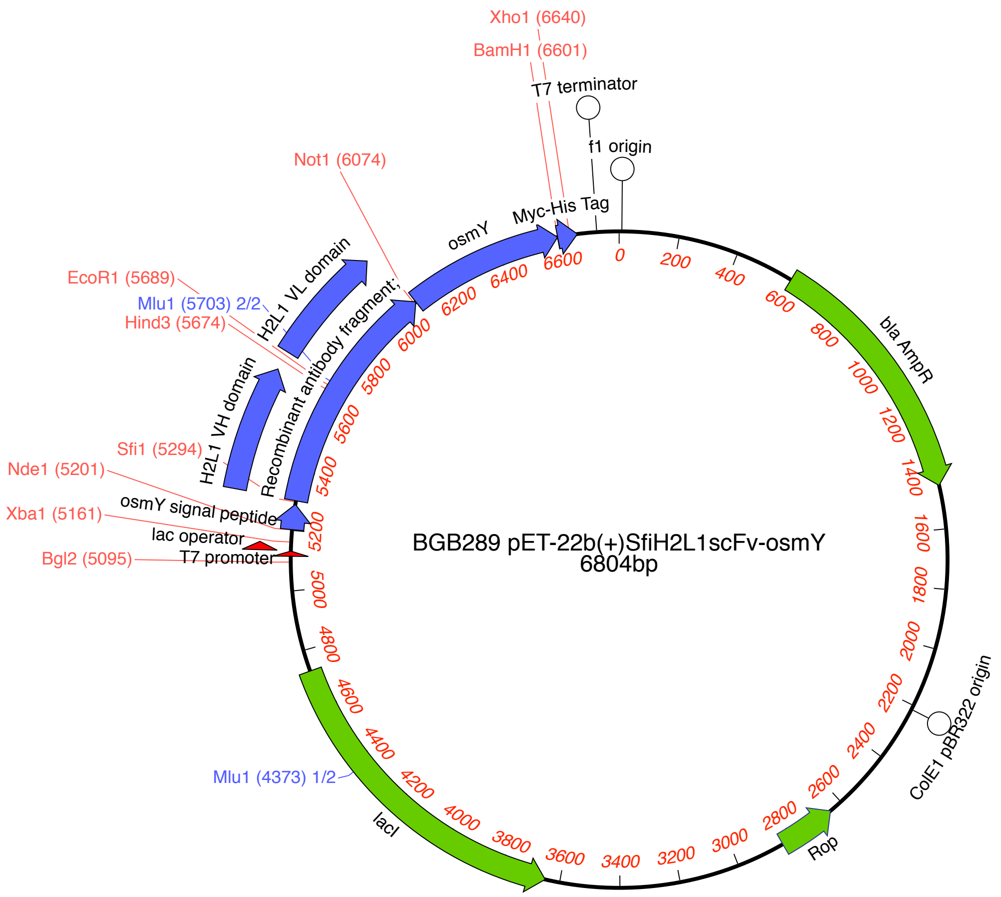

Supplementary Figure 1. Parental scFv-ompY expression vector.

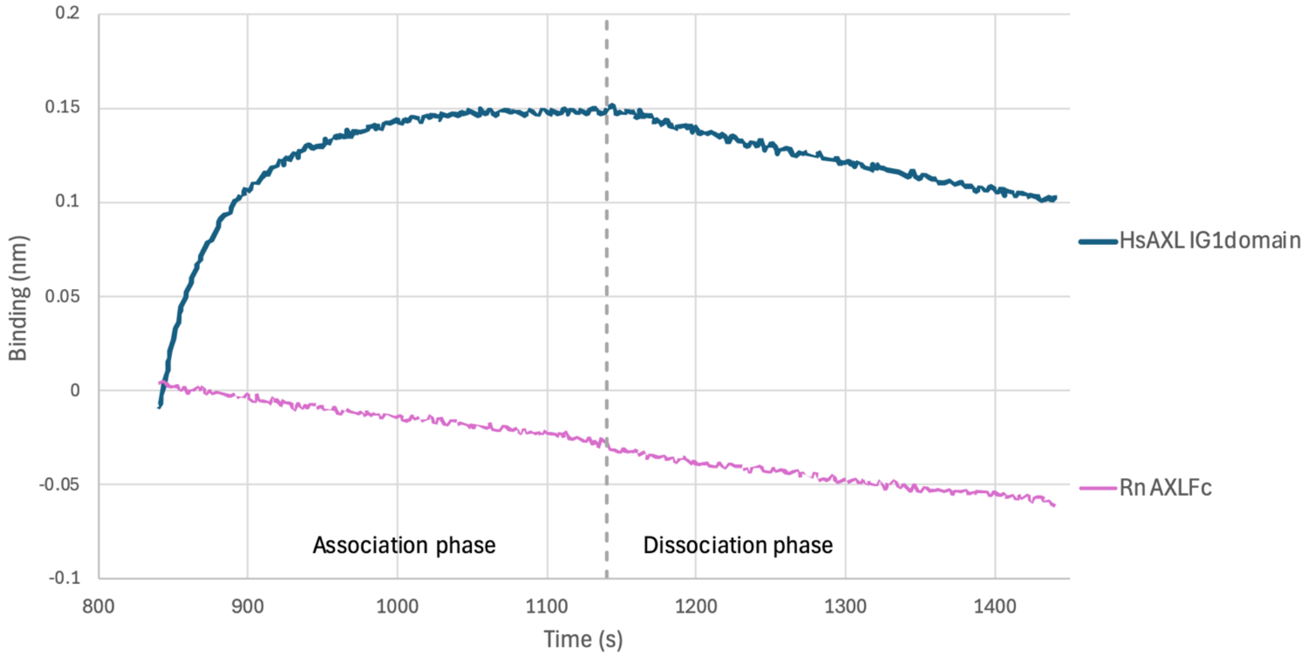

Supplementary Figure 2. BLI binding curves of human AXL IG1 domain-Fc and Rat AXL-Fc to parental scFv.

```
         1          11         21         31         41         51
AXL Ig1  EESPFVGNPG NITGARGLTG TLRCQLQVQG EPPEVHWLRD GQILELADST QTQVPLGEDE
         61         71         81         91
         QDDWIVVSQL RITSLQLSDT GQYQCLVFLG HQTFVS
```

Supplementary Figure 3. Residue numbering for the mature AXL Ig1. Amino acid positions are indicated with numbers above the sequence. Due to omission of the N-terminus signal peptide from the mature protein sequence, the residue numbering starts from position E33 of the original numbering scheme by Bryan et al.,[1] which is considered E1 in the context of this study.

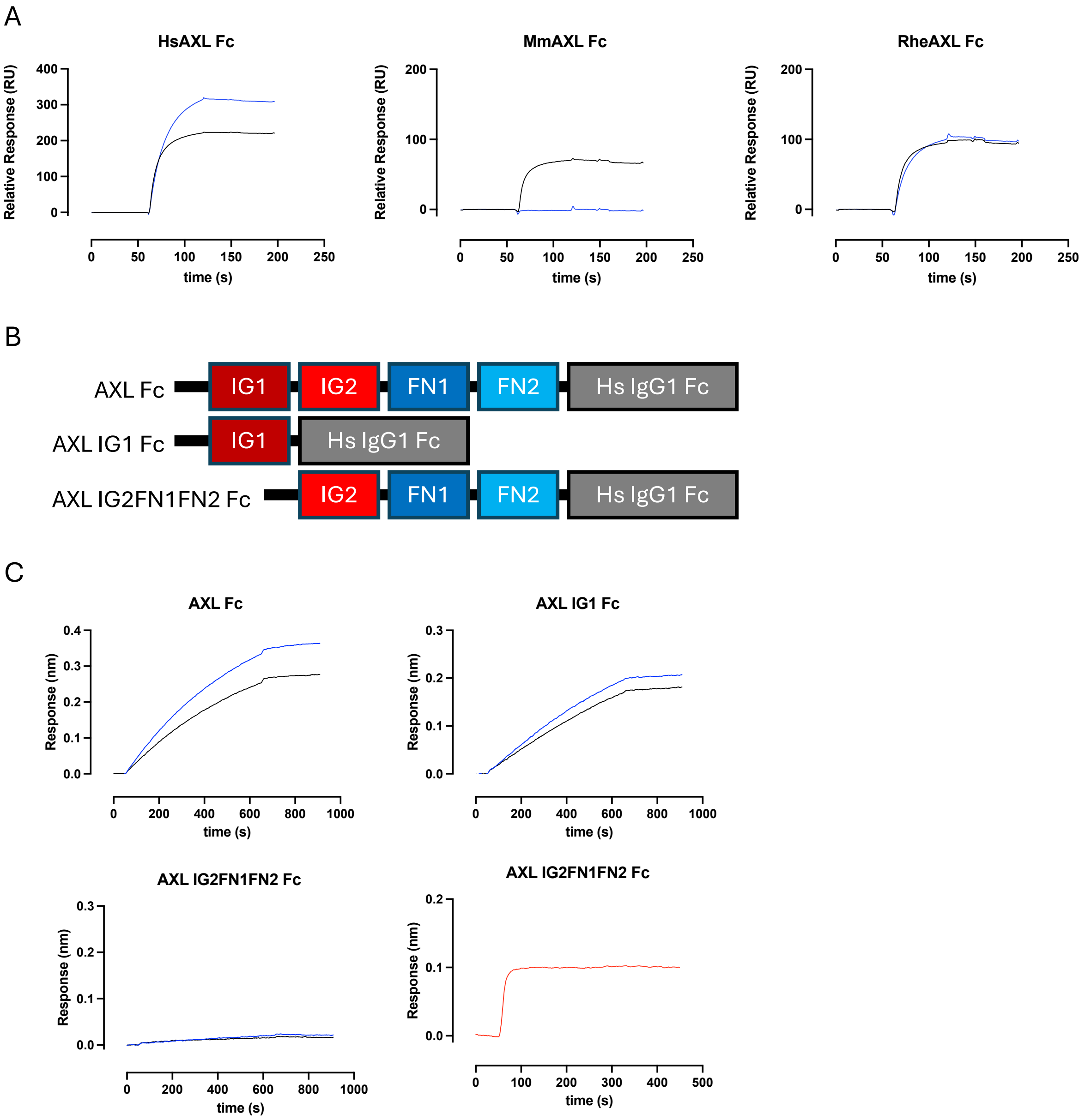

Supplementary Figure 4. Tilvestamab binds to the Ig1 domain of human AXL. (A) Surface plasmon resonance sensorgrams showing tilvestamab binding (blue) to human AXL Fc (left panel) and rhesus monkey AXL Fc (right panel) but not to mouse AXL Fc (middle panel) immobilised on a Biacore CM5 sensor chip. Binding of an anti-AXL monoclonal antibody that recognises human, rhesus monkey, and mouse AXL (YW327.6S2var)[2] is also shown as a control for the presence and integrity of the immobilised proteins (black). **(B)** Structural composition of the recombinant proteins produced to map tilvestamab epitope. **(C)** BLI sensograms of association of tilvestamab (blue) to immobilised recombinant proteins. Tilvestamab binds recombinant proteins incorporating the entire AXL extracellular domain (left upper panel) or just the Ig1 domain (right upper panel) but does not bind a protein lacking the Ig1 domain (lower panels). YW327.6S2var (black) and 1H12, an anti-AXL monoclonal antibody that binds the Ig2 domain of human AXL (red)[3] were used as controls for the presence and integrity of recombinant proteins not bound by tilvestamab.

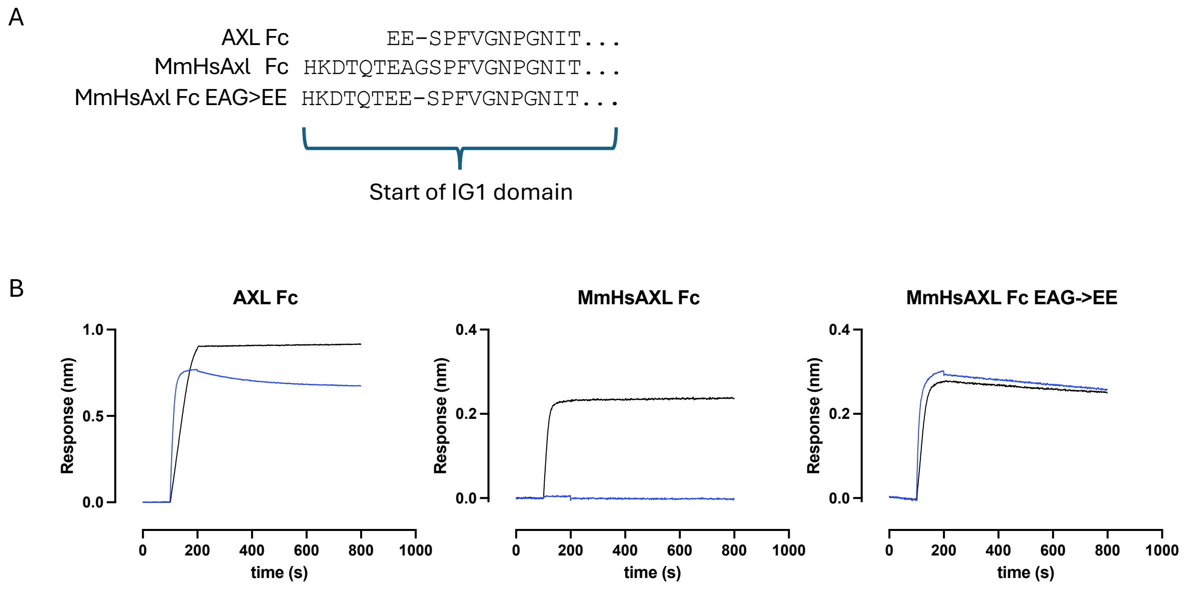

Supplementary Figure 5. A single glutamate residue is essential for tilvestamab binding to AXL. (A) Comparison of the N-terminal sequences of human AXL Fc, and mouse Axl Fc with a humanized Ig1 domain with and without mutation of the mouse N-terminal peptide from HKDTQTEAG (MmHsAxl) to HKDTQTEE (MmHsAxl Fc EAG>EE). Sequences are shown from the first amino-acid following the predicted signal peptide cleave sites for human and mouse AXL. In each case the sequence of the Ig1 domain is identical. **(B)** BLI sensograms of association of tilvestamab (blue) or YW327.6S2var (black) to immobilised recombinant proteins. Tilvestamab is unable to bind the Ig1 domain in the context of the mouse N-terminal peptide (middle panel). Mutation of the mouse sequence EAG to match the human sequence EE restores binding (right panel).

Supplementary Tables

Supplementary Table 1. Primers for generation of mutations in heavy chain CDRs

Name	Sequence
**VH CDR1**	
VHCDR1_new f	TGGGTGCGTCAGGCGCCGGGTA
VHCDR1_Parentr	GTT GAT ATA AAA GTC GGT GAA GCT GTA ACC GCT CG<br><N I Y F D T F.....
VHCDR1_N36Ar	Ggc GAT ATA AAA GTC GGT GAA GCT GTA ACC GCT CG<br><**A** I Y F D T F.....
VHCDR1_I35Ar	GTT Ggc ATA AAA GTC GGT GAA GCT GTA ACC GCT CG<br><N **A** Y F D T F.....
VHCDR1_Y34Ar	GTT GAT Agc AAA GTC GGT GAA GCT GTA ACC GCT CG<br><N I **A** F D T F.....
VHCDR1_F33Ar	GTT GAT ATA Agc GTC GGT GAA GCT GTA ACC GCT CG<br><N I Y **A** D T F.....
VHCDR1_Y34Xr	GTT GAT Akn AAA GTC GGT GAA GCT GTA ACC GCT CG<br><N I **X** F D T F.....
VHCDR1_F33Xr	GTT GAT ATA Akn GTC GGT GAA GCT GTA ACC GCT CG<br><N I Y **X** D T F.....
VHCDR1_D32Ar	GTT GAT ATA AAA GgC GGT GAA GCT GTA ACC GCT CG<br><N I Y F **A** T F.....
VHCDR1_T31Ar	GTT GAT ATA AAA GTC GGc GAA GCT GTA ACC GCT CG<br><N I Y F D **A** F.....
**Additional oligos for gap-filling:**	
VHCDR1_F33Dr	GTT GAT ATA Atc GTC GGT GAA GCT GTA ACC GCT CG
VHCDR1_F33Tr	GTT GAT ATA Agt GTC GGT GAA GCT GTA ACC GCT CG
VHCDR1_Y34Dr	GTT GAT Atc AAA GTC GGT GAA GCT GTA ACC GCT CG
VHCDR1_Y34Sr	GTT GAT Act AAA GTC GGT GAA GCT GTA ACC GCT CG
**VH CDR2 RHS**	
VHCDR2RHS_new r	GTGTTGTCACCGCCCGGGAA
VHCDR2RHS_Parent	CTA CTA TAA CGA AAA GTT CAA AGG TCG TTT TACC<br>Y Y N E K F K G R F T
VHCDR2RHS_Y60Af n	C GCC TAT AAC GAA AAG TTC AAA GGT CGT TTT ACC<br>**A** Y N E K F K G R F T
VHCDR2RHS_Y60Xf n	C NMC TAT AAC GAA AAG TTC AAA GGT CGT TTT ACC<br>**X** Y N E K F K G R F T
VHCDR2RHS_Y61Af n	C TAC GCT AAC GAA AAG TTC AAA GGT CGT TTT ACC<br>Y **A** N E K F K G R F T
VHCDR2RHS_N62Af n	C TAC TAT GCC GAA AAG TTC AAA GGT CGT TTT ACC<br>Y Y **A** E K F K G R F T
VHCDR2RHS_E63Af n	C TAC TAT AAC GCA AAG TTC AAA GGT CGT TTT ACC<br>Y Y N **A** K F K G R F T
VHCDR2RHS_K64Af n	C TAC TAT AAC GAA GCG TTC AAA GGT CGT TTT ACC<br>Y Y N E **A** F K G R F T
VHCDR2RHS_F65Af n	C TAC TAT AAC GAA AAG GCC AAA GGT CGT TTT ACC C<br>Y Y N E K **A** K G R F T
VHCDR2RHS_K66Af n	C TAC TAT AAC GAA AAG TTC GCA GGT CGT TTT ACC<br>Y Y N E K F **A** G R F T
VHCDR2RHS_G67Af n	C TAC TAT AAC GAA AAG TTC AAA GCT CGT TTT ACC<br>Y Y N E K F K **A** R F T
**Additional oligos for gap-filling:**	
VHCDR2RHS_Y60Pf	C cTC TAT AAC GAA AAG TTC AAA GGT CGT TTT ACC
**VH CDR2 LHS**	
VHCDR2LHS_r:	CGCAACCCACTCCAGACC, Tm= 68°C, Ta = 70°C
VHCDR2LHS_Parent	CGT ATT TTC CCG GGC GGT GAC AAC ACC TAC TAT AAC GA<br>R I F P G G D N T Y Y N E
VHCDR2LHS_R51Af	GCT ATT TTC CCG GGC GGT GAC AAC ACC TAC TAT AAC GA<br>**A** I F P G G D N T Y Y N E
VHCDR2LHS_I52Af	CGT GCT TTC CCG GGC GGT GAC AAC ACC TAC TAT AAC GA<br>R **A** F P G G D N T Y Y N E
VHCDR2LHS_F53Af	CGT ATT GCC CCG GGC GGT GAC AAC ACC TAC TAT AAC GA<br>R I **A** P G G D N T Y Y N E
VHCDR2LHS_P54Af	CGT ATT TTC GCG GGC GGT GAC AAC ACC TAC TAT AAC GA<br>R I F **A** G G D N T Y Y N E
VHCDR2LHS_G55Af	CGT ATT TTC CCG GCC GGT GAC AAC ACC TAC TAT AAC GA<br>R I F P **A** G D N T Y Y N E
VHCDR2LHS_G56Af	CGT ATT TTC CCG GGC GCT GAC AAC ACC TAC TAT AAC GA<br>R I F P G **A** D N T Y Y N E
VHCDR2LHS_D57Af	CGT ATT TTC CCG GGC GGT GCC AAC ACC TAC TAT AAC GA<br>R I F P G G **A** N T Y Y N E
VHCDR2LHS_N58Af	CGT ATT TTC CCG GGC GGT GAC GCC ACC TAC TAT AAC GA<br>R I F P G G D **A** T Y Y N E
VHCDR2LHS_T58Af	CGT ATT TTC CCG GGC GGT GAC AAC GCC TAC TAT AAC GA<br>R I F P G G D N **A** Y Y N E
VHCDR2LHS_R51Xf	NMT ATT TTC CCG GGC GGT GAC AAC ACC TAC TAT AAC GA<br>**X** I F P G G D N T Y Y N E
VHCDR2LHS_F53Xf	CGT ATT NMT CCG GGC GGT GAC AAC ACC TAC TAT AAC GA<br>R I **X** P G G D N T Y Y N E
**Additional oligos for gap-filling:**	
VHCDR2LHS_F53HYf	CGT ATT yaT CCG GGC GGT GAC AAC ACC TAC TAT AAC GA

Name	Sequence
**VH CDR3**	
VHCDR3_r	ACGCGCGCAATAGTAAAC, Tm=64°C, Ta=65
VHCDR3_Parentf	CGT GGC CTG TAC TAT GCG ATG GAT TAT TGG GGC CAA G<br>R G L Y Y A M D Y W G Q
VHCDR3_R100A	gcT GGC CTG TAC TAT GCG ATG GAT TAT TGG GGC CAA G<br>A G L Y Y A M D Y W G Q
VHCDR3_G101A	CGT GcC CTG TAC TAT GCG ATG GAT TAT TGG GGC CAA G<br>R A L Y Y A M D Y W G Q
VHCDR3_L102A	CGT GGC gcG TAC TAT GCG ATG GAT TAT TGG GGC CAA G<br>R G A Y Y A M D Y W G Q
VHCDR3_Y103A	CGT GGC CTG gcC TAT GCG ATG GAT TAT TGG GGC CAA G<br>R G L A Y A M D Y W G Q
VHCDR3_Y104A	CGT GGC CTG TAC gcT GCG ATG GAT TAT TGG GGC CAA G<br>R G L Y a A M D Y W G Q
VHCDR3_M106A	CGT GGC CTG TAC TAT GCG gcg GAT TAT TGG GGC CAA G<br>R G L Y Y A A D Y W G Q
VHCDR3_D107A	CGT GGC CTG TAC TAT GCG ATG gcT TAT TGG GGC CAA G<br>R G L Y Y A M A Y W G Q
VHCDR3_Y108A	CGT GGC CTG TAC TAT GCG ATG GAT gcT TGG GGC CAA G<br>R G L Y Y A M D A W G Q
VHCDR3_R100X	NMT GGC CTG TAC TAT GCG ATG GAT TAT TGG GGC CAA G<br>X G L Y Y A M D Y W G Q
VHCDR3_L102X	CGT GGC NMT TAC TAT GCG ATG GAT TAT TGG GGC CAA G<br>R G X Y Y A M D Y W G Q
VHCDR3_Y103X	CGT GGC CTG NMT TAT GCG ATG GAT TAT TGG GGC CAA G<br>R G L X Y A M D Y W G Q
VHCDR3_Y104X	CGT GGC CTG TAC NMT GCG ATG GAT TAT TGG GGC CAA G<br>R G L Y X A M D Y W G Q
**Additional oligos for gap-filling:**	
VHCDR3_R100Y	taT GGC CTG TAC TAT GCG ATG GAT TAT TGG GGC CAA G
VHCDR3_Y103N	CGT GGC CTG aaT TAT GCG ATG GAT TAT TGG GGC CAA G
VHCDR3_Y103ST	CGT GGC CTG wcT TAT GCG ATG GAT TAT TGG GGC CAA G
VHCDR3_Y104P	CGT GGC CTG TAC ccT GCG ATG GAT TAT TGG GGC CAA G

Supplementary Table 2. Primers for generation of mutations in light chain CDRs

Name	Sequence
**VL CDR1 LHS**	
VLCDR1LHS_r	GCAGGTGATGGTAACACGATC, Tm=66°C, Ta=67
VLCDR1LHS_Parent f	CGT AGC AGC CAG AGC CTG GTT CAC AGC AAC GGT A<br>R S S Q S L V H S N G
VLCDR1LHS_R161A	gcT AGC AGC CAG AGC CTG GTT CAC AGC AAC GGT A<br>**A** S S Q S L V H S N G
VLCDR1LHS_S162A	CGT gcC AGC CAG AGC CTG GTT CAC AGC AAC GGT A<br>R **A** S Q S L V H S N G
VLCDR1LHS_S163A	CGT AGC gcC CAG AGC CTG GTT CAC AGC AAC GGT A<br>R S **A** Q S L V H S N G
VLCDR1LHS_Q164A	CGT AGC AGC GcG AGC CTG GTT CAC AGC AAC GGT A<br>R S S **A** S L V H S N G
VLCDR1LHS_S165A	CGT AGC AGC CAG gcC CTG GTT CAC AGC AAC GGT A<br>R S S Q **A** L V H S N G
VLCDR1LHS_L166A	CGT AGC AGC CAG AGC gcG GTT CAC AGC AAC GGT A<br>R S S Q S **A** V H S N G
VLCDR1LHS_V167A	CGT AGC AGC CAG AGC CTG GcT CAC AGC AAC GGT A<br>R S S Q S L **A** H S N G
VLCDR1LHS_H168A	CGT AGC AGC CAG AGC CTG GTT gcC AGC AAC GGT A<br>R S S Q S L V **A** S N G
VLCDR1LHS_H168X	CGT AGC AGC CAG AGC CTG GTT NMT AGC AAC GGT A<br>R S S Q S L V **X** S N G
Additional oligos for gap-filling:	
VLCDR1LHS_H168ST	CGT AGC AGC CAG AGC CTG GTT wcT AGC AAC GGT A
**VL CDR1 RHS**	
VLCDR1RHS_r	GTGAACCAGGCTCTGGCT, Tm=67°C, Ta=64
VLCDR1RHS_Parent f	AGC AAC GGT ATT CCG TAC CTG CAC TGG TAT CAG C<br>S N G I P Y L H W Y Q
VLCDR1RHS_S169A	gcC AAC GGT ATT CCG TAC CTG CAC TGG TAT CAG C<br>**A** N G I P Y L H W Y Q
VLCDR1RHS_N170A	AGC gcC GGT ATT CCG TAC CTG CAC TGG TAT CAG C<br>S **A** G I P Y L H W Y Q
VLCDR1RHS_G171A	AGC AAC GcT ATT CCG TAC CTG CAC TGG TAT CAG C<br>S N **A** I P Y L H W Y Q
VLCDR1RHS_I172A	AGC AAC GGT gcT CCG TAC CTG CAC TGG TAT CAG C<br>S N G **A** P Y L H W Y Q
VLCDR1RHS_P173A	AGC AAC GGT ATT gcG TAC CTG CAC TGG TAT CAG C<br>S N G I **A** Y L H W Y Q
VLCDR1RHS_Y174A	AGC AAC GGT ATT CCG gcC CTG CAC TGG TAT CAG C<br>S N G I P **A** L H W Y Q
VLCDR1RHS_L175A	AGC AAC GGT ATT CCG TAC gcG CAC TGG TAT CAG C<br>S N G I P Y **A** H W Y Q
VLCDR1RHS_H176A	AGC AAC GGT ATT CCG TAC CTG gcC TGG TAT CAG C<br>S N G I P Y L **A** W Y Q
VLCDR1RHS_Y174X	AGC AAC GGT ATT CCG NMT CTG CAC TGG TAT CAG C<br>S N G I P **X** L H W Y Q
Additional oligos for gap-filling:	
VLCDR1RHS_Y174HP	AGC AAC GGT ATT CCG cmT CTG CAC TGG TAT CAG C
VLCDR1RHS_Y174ST	AGC AAC GGT ATT CCG wcT CTG CAC TGG TAT CAG C
**VL CDR2**	
VLCDR2_r (new)	GATCAGCAGCTTCGGCGCTTTG, Tm=67°C, Ta=70°C
VLCDR2_Parent f	TAC CGT GTG AGC AAC CGT TTC AGC GGT GTT CCG A<br>Y R V S N R F S G V P
VLCDR2_Y191A	gcC CGT GTG AGC AAC CGT TTC AGC GGT GTT CCG A<br>**A** R V S N R F S G V P
VLCDR2_R192A	TAC gcT GTG AGC AAC CGT TTC AGC GGT GTT CCG A<br>Y **A** V S N R F S G V P
VLCDR2_V193A	TAC CGT GcG AGC AAC CGT TTC AGC GGT GTT CCG A<br>Y R **A** S N R F S G V P
VLCDR2_S194A	TAC CGT GTG gcC AAC CGT TTC AGC GGT GTT CCG A<br>Y R V **A** N R F S G V P
VLCDR2_N195A	TAC CGT GTG AGC gcC CGT TTC AGC GGT GTT CCG A<br>Y R V S **A** R F S G V P
VLCDR2_R196A	TAC CGT GTG AGC AAC gcT TTC AGC GGT GTT CCG A<br>Y R V S N **A** F S G V P
VLCDR2_F197A	TAC CGT GTG AGC AAC CGT gcC AGC GGT GTT CCG A<br>Y R V S N R **A** S G V P
VLCDR2_S198A	TAC CGT GTG AGC AAC CGT TTC gcC GGT GTT CCG A<br>Y R V S N R F **A** G V P
VLCDR2_Y191X	NMT CGT GTG AGC AAC CGT TTC AGC GGT GTT CCG A<br>**X** R V S N R F S G V P
VLCDR2_R192X	TAC NMT GTG AGC AAC CGT TTC AGC GGT GTT CCG A<br>Y **X** V S N R F S G V P
VLCDR2_N195X	TAC CGT GTG AGC NMT CGT TTC AGC GGT GTT CCG A<br>Y R V S **X** R F S G V P
Additional oligos for gap-filling:	
VLCDR2_Y191DHN	vaT CGT GTG AGC AAC CGT TTC AGC GGT GTT CCG A
VLCDR2_R192A	TAC gcT GTG AGC AAC CGT TTC AGC GGT GTT CCG A
VLCDR2_R192H	TAC caT GTG AGC AAC CGT TTC AGC GGT GTT CCG A
VLCDR2_N195PT	TAC CGT GTG AGC mcT CGT TTC AGC GGT GTT CCG A
VLCDR2_N195Y	TAC CGT GTG AGC taT CGT TTC AGC GGT GTT CCG A

Name	Sequence
**VL CDR3**	
VLCDR3_f	GCAATAGTAGGTCGCAAAATCC,6 Tm=4°C, Ta=65°C
VLCDR3_Parent f	AGC CAG GGT ACC CAT GTG CCG CCG ACC TTC GGT CAA G<br>S Q G T H V P P T F G Q
VLCDR3_S231A	gcC CAG GGT ACC CAT GTG CCG CCG ACC TTC GGT CAA G<br>**A** Q G T H V P P T F G Q
VLCDR3_Q232A	AGC gcG GGT ACC CAT GTG CCG CCG ACC TTC GGT CAA G<br>S **A** G T H V P P T F G Q
VLCDR3_G233A	AGC CAG GcT ACC CAT GTG CCG CCG ACC TTC GGT CAA G<br>S Q **A** T H V P P T F G Q
VLCDR3_T234A	AGC CAG GGT gcC CAT GTG CCG CCG ACC TTC GGT CAA G<br>S Q G **A** H V P P T F G Q
VLCDR3_H235A	AGC CAG GGT ACC gcT GTG CCG CCG ACC TTC GGT CAA G<br>S Q G T **A** V P P T F G Q
VLCDR3_V236A	AGC CAG GGT ACC CAT GcG CCG CCG ACC TTC GGT CAA G<br>S Q G T H **A** P P T F G Q
VLCDR3_P237A	AGC CAG GGT ACC CAT GTG gCG CCG ACC TTC GGT CAA G<br>S Q G T H V **A** P T F G Q
VLCDR3_P238A	AGC CAG GGT ACC CAT GTG CCG gCG ACC TTC GGT CAA G<br>S Q G T H V P **A** T F G Q
VLCDR3_T239A	AGC CAG GGT ACC CAT GTG CCG CCG gCC TTC GGT CAA G<br>S Q G T H V P P **A** F G Q
VLCDR3_G233X	AGC CAG NMT ACC CAT GTG CCG CCG ACC TTC GGT CAA G<br>S Q **X** T H V P P T F G Q
VLCDR3_P238X	AGC CAG GGT ACC CAT GTG CCG NMT ACC TTC GGT CAA G<br>S Q G T H V P **X** T F G Q
Additional oligos for gap-filling:	
VLCDR3_G233T	AGC CAG acT ACC CAT GTG CCG CCG ACC TTC GGT CAA G
VLCDR3_P238S	AGC CAG GGT ACC CAT GTG CCG tcT ACC TTC GGT CAA G

Supplementary References

1 O'Bryan, J. P. *et al.* axl, a transforming gene isolated from primary human myeloid leukemia cells, encodes a novel receptor tyrosine kinase. *Mol Cell Biol* **11**, 5016-5031 (1991). https://doi.org/10.1128/mcb.11.10.5016-5031.1991

2 Ye, X. *et al.* An anti-Axl monoclonal antibody attenuates xenograft tumor growth and enhances the effect of multiple anticancer therapies. *Oncogene* **29**, 5254-5264 (2010). https://doi.org/10.1038/onc.2010.268

3 Ahmed, L. *et al.* Novel anti-human Axl monoclonal antibodies for improved patient biomarker studies. *Diagnostic Pathology* **2** (2016). https://doi.org/10.17629/www.diagnosticpathology.eu-2016-2:104
